# The distribution of monolignol glucosides coincides with lignification during the formation of compression wood in *Pinus thunbergii*

**DOI:** 10.1111/tpj.17209

**Published:** 2024-12-14

**Authors:** Naoki Maeda, Dan Aoki, Syunya Fujiyasu, Yasuyuki Matsushita, Masato Yoshida, Hideto Hiraide, Hayato Mitsuda, Yuki Tobimatsu, Kazuhiko Fukushima

**Affiliations:** ^1^ Graduate School of Bioagricultural Sciences Nagoya University Nagoya 464‐8601 Japan; ^2^ Institute of Agriculture Tokyo University of Agriculture and Technology Tokyo 183‐8509 Japan; ^3^ Graduate School of Agriculture Kyoto University Kitashirakawa‐oiwakecho Kyoto 606‐8502 Japan; ^4^ Research Institute for Sustainable Humanosphere Kyoto University Gokasho Uji 611‐0011 Japan

**Keywords:** compression wood, lignification, cell wall, frozen hydrated, secondary ion mass spectrometry, *Pinus thunbergii*, fluorescence probes, chemical mapping, imaging mass spectrometry

## Abstract

The distributions of monolignol glucosides (MLGs) in compression and opposite woods of *Pinus thunbergii* were assessed using cryo‐time‐of‐flight secondary ion mass spectrometry to investigate their involvement in lignification. *p*‐Glucocoumaryl alcohol (PG) was identified in the region of the differentiating xylem adjacent to the cambial zone only in compression wood, whereas coniferin (CF) was similarly localized in both compression and opposite woods. Their distribution from the phloem to the xylem was evaluated by high‐performance liquid chromatography (HPLC) using serial tangential sections. Variations in storage amounts of CF and PG in the stem of *P. thunbergii* agreed with lignification stages of the tracheid, supporting the idea that MLGs act as a storage and transportation form of lignin precursors. The imaging of monolignol (ML)‐dependent active lignification sites using fluorescence‐tagged MLs supported distinct distribution patterns of MLGs for lignification in compression and opposite woods. Methylation–thioacidolysis was applied to compression and opposite wood samples to examine the structural difference between the guaiacyl (G) and *p*‐hydroxyphenyl (H) units in lignin. Most of the H units in compression wood were detected as lignin end groups via thioacidolysis. PG was detected in opposite wood by HPLC; however, the H unit was not detected by thioacidolysis. The differences in ML and MLG distributions, enzyme activity, and resultant lignin structures between the G and H units suggest the possibility of individual mechanisms regulating the heterogeneous structures of G and H unit in lignin.

## Significance Statement

The microscopic distribution of coniferin and p‐glucocoumaryl alcohol as monolignol precursors in the differentiating xylem region of *Pinus thunbergii* compression wood was visualized by imaging mass spectrometry using frozen‐hydrated samples. The differences in monolignols and monolignol glucosides distributions, enzyme activity, and resultant lignin structures between the G and H units suggest the possibility of individual mechanisms regulating the heterogeneous structures of G and H unit in lignin.

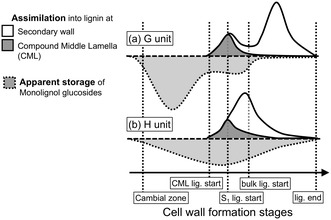

## INTRODUCTION

Lignin is a major component of woody plant cell walls, and is synthesized via oxidative coupling of monolignols (MLs) and related compounds (Boerjan et al., 2003; Meents et al., 2018; Tobimatsu & Schuetz, 2019; Wang et al., 2013; Weng & Chapple, 2010; Whetten et al., 1998). The basic structural units of lignin are guaiacyl (G) units derived from coniferyl alcohol (CA). In addition, syringyl (S) and *p*‐hydroxyphenyl (H) units are formed from sinapyl alcohol and *p*‐coumaryl alcohol (PA), respectively. The ratio of these units varies with plant species and tissues. For example, gymnosperm lignin mainly consists of G‐unit and a small number of H‐unit in compression wood. In angiosperm lignin, S‐unit is more abundant than G‐unit; however, the ratio depends on the species. The chemical composition of lignin has a significant influence on the physical and chemical properties of the cell wall.

Mechanisms underlying lignin biosynthesis have long been studied. Lignin deposition in the multilayered plant cell wall can be microscopically observed using ultraviolet (UV) light (Fergus et al., 1969; Takabe et al., 1981; Wardrop, 1957). The lignification process in the tracheid comprising the gymnosperm xylem is generally divided into three stages. First, lignification starts in the cell corners or middle lamellar regions simultaneously with outermost (S_1_) layer formation in the secondary wall domain. Next, the primary wall and S_1_ layers are slowly lignified during the formation of the middle (S_2_) and inner (S_3_) layers of the secondary wall. Finally, most active lignification, also called “bulk” lignification, occurs in entire secondary wall regions after formation of the S_3_ layer. In compression wood, which is a specific tissue in gymnosperms formed to adjust the orientation of axial growth, a lignin‐rich layer (S_2_L) is observed in the S_2_ layer. Furthermore, the S_3_ layer is lost (Donaldson, 2001; Donaldson et al., 1999; Wagner et al., 2012).

A central subject in the discussion of lignification is the precursors and related compounds. MLs, the direct precursors of lignin, have a reactive phenolic hydroxyl group and low water solubility. Therefore, the suitability of these materials for storage and transportation is limited. However, the glucosylated forms of MLs, namely monolignol glucosides (MLGs), such as coniferin (CF), syringin, and *p*‐glucocoumaryl alcohol (PG), are often found stored in plants, and their involvement in lignification has been studied. MLGs are less toxic, more stable, and more water‐soluble than MLs (Dharmawardhana et al., 1995; Freudenberg et al., 1955; Freudenberg & Harkin, 1963; Jones & Vogt, 2001; Samuels et al., 2002; Terashima et al., 2016; Wang et al., 2013; Whetten et al., 1998; Whetten & Sederoff, 1995). Therefore, MLGs have been proposed to play an essential role in lignification as storage and transportation forms of the precursor MLs (Freudenberg & Harkin, 1963; Fukushima et al., 1996, 1997; Savidge, 1989; Terazawa et al., 1984; Tsuyama et al., 2013, 2019).

Several studies have questioned the role of MLGs as direct intermediates for lignification, at least in the eudicot non‐wood model plant *Arabidopsis* (Barros et al., 2015; Lanot et al., 2006; Le Roy et al., 2016; Meents et al., 2018; Perkins et al., 2019). *Arabidopsis* mutants with downregulated ML glucosylation by UDP‐glucose transferases (UGT72E1–E3, which can glucosylate several phenylpropanoids) stay alive (Lanot et al., 2006). However, a considerable amount of data supports their role as intermediates for lignification in several woody plants including softwood (Aoki et al., 2016; Miyagawa et al., 2020; Morikawa et al., 2010; Samuels et al., 2002; Savidge, 1989; Savidge & Förster, 2001; Terashima & Fukushima, 1988; Tsuji et al., 2005; Tsuyama & Takabe, 2014), hardwood (Aoki et al., 2019; Miyagawa et al., 2020; Tsuyama et al., 2019; Tsuyama & Takabe, 2014, 2015), and woody grass (e.g., bamboo. Miyagawa et al., 2020) species. Furthermore, regarding the assimilation behavior of MLGs into cell‐wall lignin, administration experiments using isotope‐labeled MLGs at aglycon moieties have been conducted to clarify the role of MLGs in lignification (Eglinton et al., 2000; Fukushima & Terashima, 1990, 1991a, 1991b; Fukushima et al., 1996, 1997; Terashima & Fukushima, 1988; Terashima et al., 2002, 2009, 2016; Tsuji et al., 2005; Xie, Robert, & Terashima, 1994; Xie, Yasuda, & Terashima, 1994). The aglycon moieties of MLGs are assimilated into cell wall lignin, suggesting that lignifying cells in the differentiating xylem region can consume MLGs as lignin precursors.

To determine the role of MLGs as a lignin precursor, analyzing the microscopic distribution of endogenous MLGs in plants is an essential but challenging. Early studies have used serial tangential sections to quantify their amounts and roughly estimate their distribution in plants using high‐performance liquid chromatography (HPLC) (Fukushima et al., 1997). Recent advances in chemical imaging techniques, such as Raman microscopy and matrix‐assisted laser desorption/ionization mass spectrometric imaging, have enabled visualizing the microscopic distribution of CF (Morikawa et al., 2010; Yoshinaga et al., 2015). These pioneering studies indicated that MLGs are stored around the differentiating xylem region, further supporting the view that they are used as a lignin precursor. However, detailed and precise imaging analysis of MLGs *in planta* are necessary because earlier imaging techniques still suffer from the potential loss of positional information of MLGs in living wood tissues owing to the relatively severe pretreatment processes for sample preparations (Yamagishi et al., 2021).

Imaging mass spectrometry using freeze‐fixed samples may be a highly promising technique for visualizing the *in planta* distribution of water‐soluble compounds such as MLs and MLGs (Aoki et al., 2018, 2022). Recently, the distributions of endogenous CF in *Ginkgo biloba* (Aoki et al., 2016) and endogenous syringin in *Syringa vulgaris* (Aoki et al., 2019) have been freeze‐fixed and visualized using cryo‐time‐of‐flight secondary ion mass spectrometry/scanning electron microscopy (cryo‐TOF‐SIMS/SEM). The distribution of CF in *G. biloba* stems has been consistent with the lignification stages of the tracheid. The apparent amount of stored CF increased from the cambial zone to the differentiating xylem region and diminished immediately after the start of bulk lignification. Syringin is widely distributed in *S. vulgaris* stem, and the most abundant region is the phloem, as previously reported for *S. vulgaris* (Kurkin et al., 1992) and *Magnolia kobus* (Fukushima et al., 1996; Okumura et al., 2017). In the differentiating xylem region, the distribution of syringin is similar to that of CF in *G. biloba*; therefore, syringin may also function as a storage and transportation form of sinapyl alcohol in the differentiating xylem region.

The ratio of structural units of lignin is controlled and correlates with the physicochemical properties of the plant cell wall. Typical gymnosperm compression woods synthesize lignin containing H units, in addition to the G unit that dominates lignin produced in normal woods (Donaldson, 2001; Lapierre et al., 1988; Lapierre & Roland, 1988; Önnerud, 2003; Timell, 1986; Zhang et al., 2017). PG is significantly stored in compression wood; however, its storage is considerably less in normal wood of *P. thunbergii*. Furthermore, an alkaline nitrobenzene oxidation method has detected a considerable number of H units in lignin of compression wood (Fukushima et al., 1997). Therefore, PG participate may in lignification as a lignin precursor, that is, as a storage and transportation form of PA, similar to CF and syringin. However, the detailed *in planta* distribution of PG has not yet been visualized. To discuss the detailed mechanism of lignification in the formation of the cell walls of compression wood, the difference between the G and H units should be examined.

In this study, *P. thunbergii* seedlings were grown under tilted conditions, and the lower and upper sides were used as the compression and opposite wood samples, respectively. The distributions of CF and PG in compression and opposite woods of *P. thunbergii* were investigated. Mono‐ and disaccharides were quantified. Because their molecular weights are nearly equal to those for MLs and MLGs, their quantitative distributions are essential information for detailed discussion using imaging mass spectrometry. Furthermore, the oxidative activity of cell‐wall‐localized enzymes was examined, and the differences in the chemical structures of lignin were investigated.

## RESULTS AND DISCUSSION

### Radial quantitative distribution of MLs, MLGs, and saccharides

**Figure 1:**
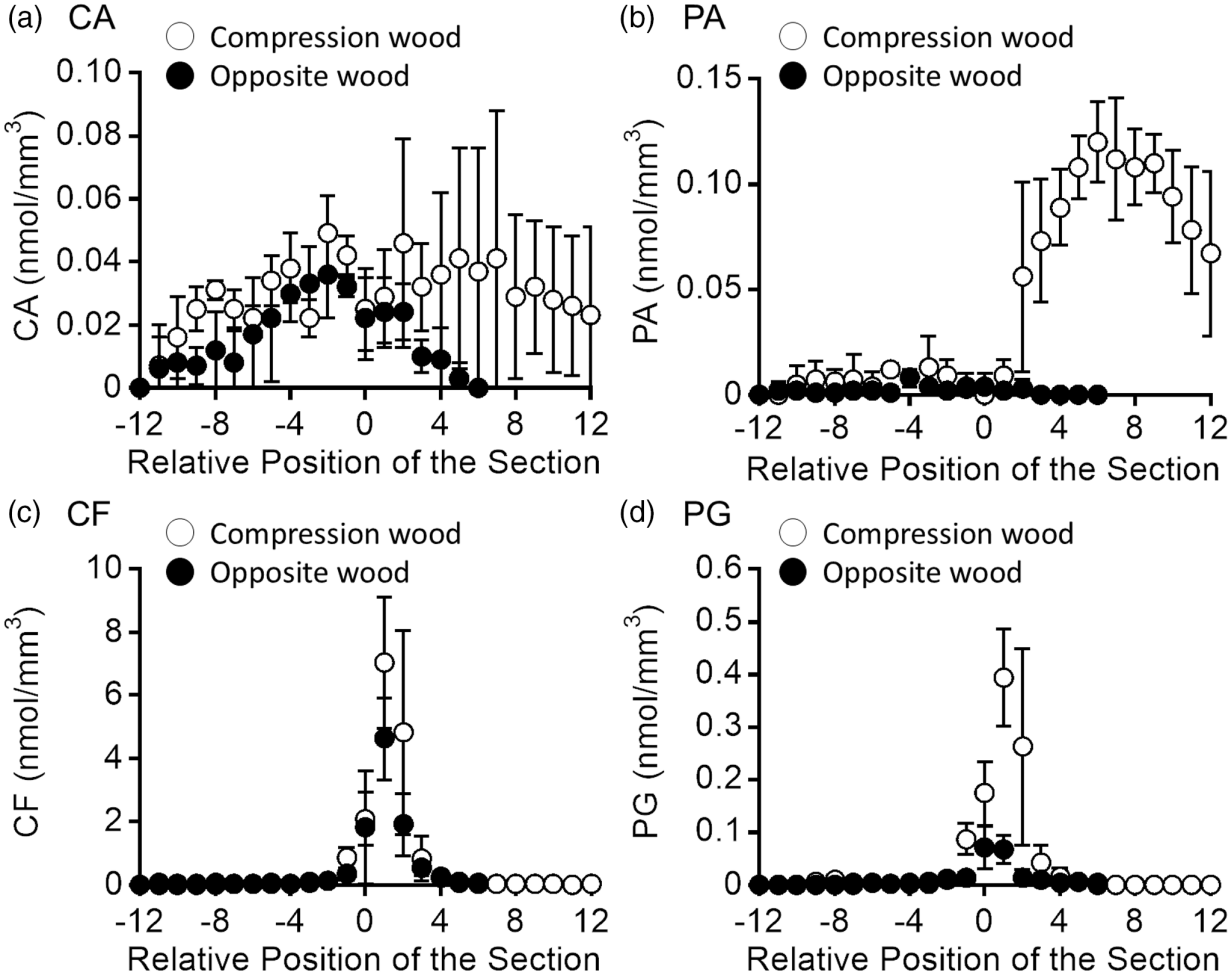
Radial distribution of (a) coniferyl alcohol (CA), (b) *p*‐coumaryl alcohol (PA), (c) coniferin (CF), and (d) *p*‐glucocoumaryl alcohol (PG) in compression and opposite woods of *Pinus thunbergii*, evaluated by high‐performance liquid chromatography (HPLC). The numbers of the cambial zone‐containing sections were determined by variation in dry weights of the sections, and the relative position of the cambial zone containing section was fixed as 0. Relative position of the section corresponds to phloem region (minus) and xylem region (plus). The means and standard deviations for each section were determined from three sets of measurements using individual sample blocks cut from the same disk.

Freeze‐fixed *P. thunbergii* stem blocks were cut into serial tangential sections (50 μm × 3 thickness for each section number) from the bark to the xylem and extracted for quantification of MLs and MLGs by HPLC. The results obtained for the compression and opposite wood samples are shown in Figure 1. The numbers of sections containing the cambial zone was determined based on the variation in dry weights of the sections (Figure S2). The relative position of the cambial zone‐containing section was fixed as 0. Relative position of the section corresponds to the phloem region (minus) and xylem region (plus).

**Figure 2:**
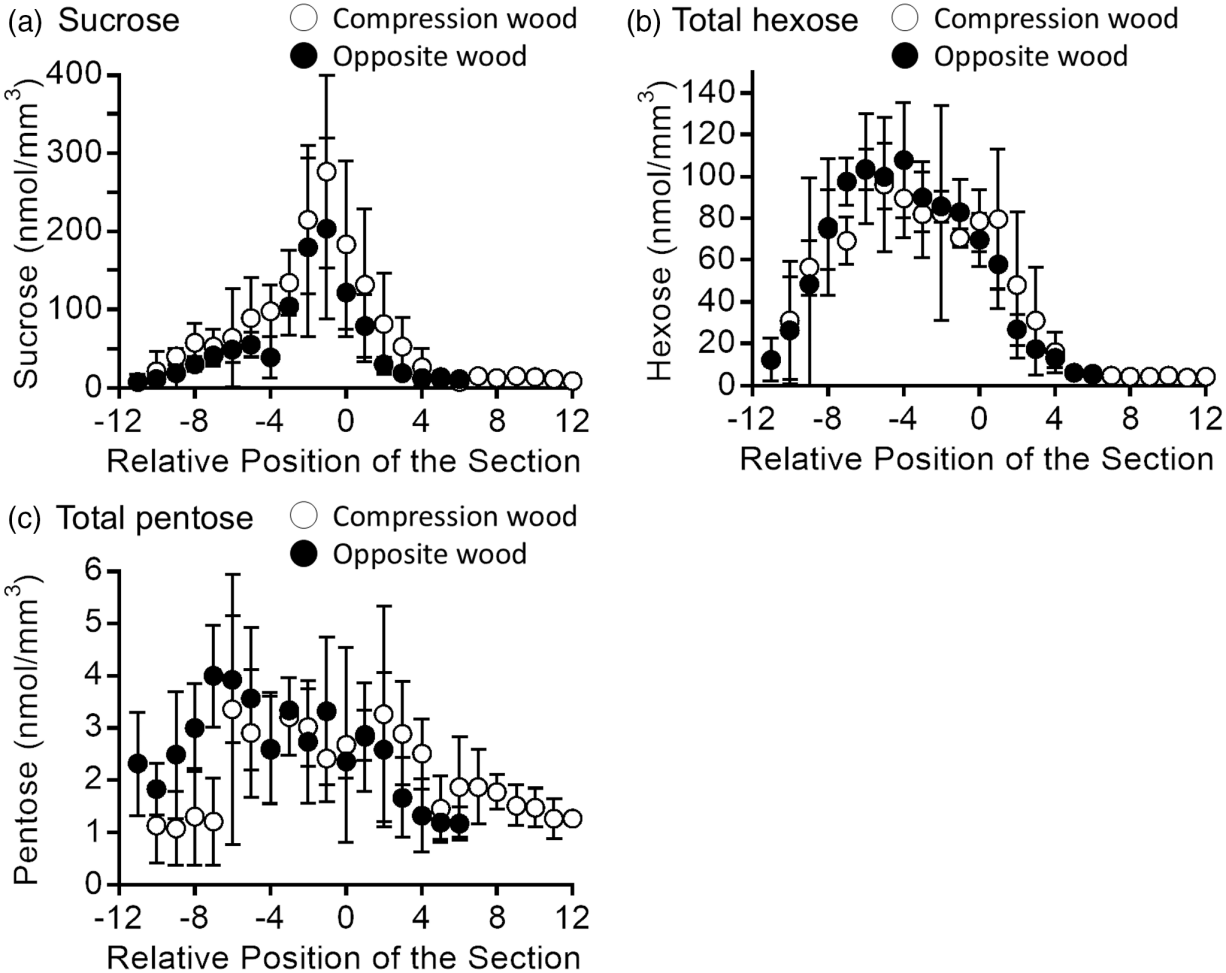
Radial distribution of (a) sucrose, (b) hexoses, and (c) pentoses in compression and opposite woods of *Pinus thunbergii*, evaluated by ion exchange chromatography (IEC). The numbers of the cambial zone‐containing sections were determined by the variation in dry weights of the sections, and the relative position of the cambial zone containing section was fixed as 0. Relative position of the section corresponds to phloem region (minus) and xylem region (plus). The means and standard deviations for each section were calculated from three sets of measurements using individual sample blocks cut from the same disk.

In opposite wood, the amount of CA (Figure 1a) and PA (Figure 1b) was small (~ 0.04 nmol mm^−3^) and similar within the measured regions. However, in compression wood, the amount of PA (Figure 1b) showed a significant increase in the xylem region (*P* < 0.01 in Student's *t*‐test, between relative positions −11–1 and section 2–12), whereas the amount of CA remained similar between the phloem and xylem regions (*P* > 0.1, between relative positions −11–1 and section 2–12). In contrast, significant amounts of CF (Figure 1c) and PG (Figure 1d) were detected specifically in the differentiating xylem region adjacent to the cambial zone in both the compression and opposite woods (*P* < 0.1, between relative positions −1 and 1, 1 and 3). The amount of PG was more significant in compression wood than in opposite wood (*P* < 0.01 between relative positions 0–2 of compression and opposite woods). Furthermore, the amount of CF (~ 8 nmol mm^−3^) was much higher than that of PG (~0.4 nmol mm^−3^). The radial distribution of saccharides was evaluated using IEC measurements. The results obtained for the compression and opposite wood samples are shown in Figure 2. In this study, sucrose, a disaccharide (C_12_H_22_O_11_), glucose, fructose, and mannose as monosaccharides of hexose (C_6_H_12_O_6_), and arabinose and xylose as monosaccharides of pentose (C_5_H_10_O_5_) were evaluated (Figure S3).

Sucrose (Figure 2a, ~300 nmol mm^−3^) was the most abundant saccharide in both compression and opposite woods. A large amount of sucrose was detected in the phloem region adjacent to the cambial zone (relative position −1). Hexoses were widely detected in the phloem region. The total amount of hexoses (Figure 2b, ~120 nmol mm^−3^) was comparable with that of sucrose and was much higher than that of MLs (Figure 1a,b, ~0.15 nmol mm^−3^), MLGs (Figure 1c,d, ~10 nmol mm^−3^), and pentoses (Figure 2c, ~5 nmol mm^−3^). Pentoses were distributed mainly in the phloem region.

### Secondary ions for cryo‐TOF‐SIMS visualization of MLGs

In TOF‐SIMS measurements, secondary ion generation is affected by the chemical conditions surrounding the target compounds. Because of the matrix effect, specific attention is necessary for determining the characteristic secondary ions to visualize the target compounds, particularly for measuring frozen‐hydrated biomaterials containing various compounds. Aqueous KCl solution of the standard compounds helps to obtain standard spectra in cryo‐TOF‐SIMS for plant tissue samples (Aoki et al., 2016, 2019, 2022; Okumura et al., 2017; Yu et al., 2022) because K^+^ and Cl^−^ are the most abundant inorganic cation and anion found in typical plant tissue samples (Broadley et al., 2012; Kirkby, 2012).

**Figure 3:**
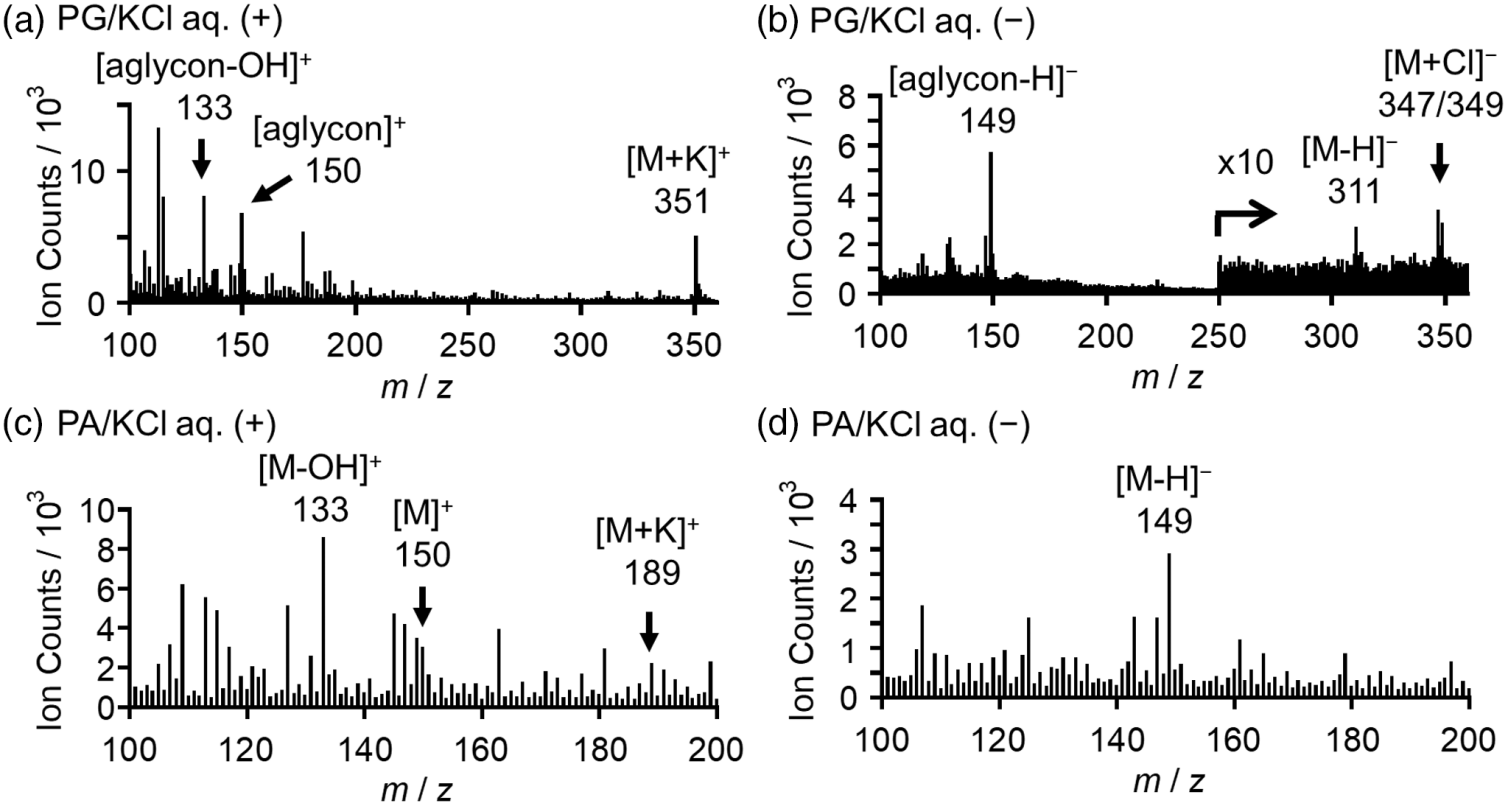
Cryo‐time‐of‐flight secondary ion mass spectrometry (Cryo‐TOF‐SIMS) (a, c) positive and (b, d) negative spectra of (a, b) PG and (c, d) PA dissolved in KCl aq.

Figure 3 shows the cryo‐TOF‐SIMS spectra of PG and PA in aqueous KCl. The cryo‐TOF‐SIMS spectra of other MLs, MLGs, and saccharides have been previously reported (Aoki et al., 2016, 2019; Okumura et al., 2017) and are summarized in Figures S4, S5, and S6, respectively. The resultant characteristic secondary ions are summarized in Table 1 and Table S1.

**Table 1:** Cryo‐TOF‐SIMS major ions for MLs, MLGs, and saccharides

Compounds	*m*/*z*	
	Positive mode	Negative mode
PA	[M−OH]^+^ 133, [M]^+^ 150, [M+K]^+^ 189	[M−H]^−^ 149
CA	[M−OH]^+^ 163, [M]^+^ 180, [M+K]^+^ 219	[M−H]^−^ 179
PG	[aglycon]^+^ 150, [M+K]^+^ 351	[aglycon−H]^−^ 149, [M−H]^−^ 311, [M+Cl]^−^ 347
CF	[aglycon]^+^ 180, [M+K]^+^ 381	[aglycon−H]^−^ 179, [M−H]^−^ 341, [M+Cl]^−^ 377
Pentose	[M+K]^+^ 189	[M−H]^−^ 149, [M+Cl]^−^ 185
Hexose	[M+K]^+^ 219	[M−H]^−^ 179, [M+Cl]^−^ 215
Sucrose	[M+K]^+^ 381	[M−H]^−^ 341, [M+Cl]^−^ 377

In the positive‐mode spectrum of PG (Figure 3a), the ion at *m*/*z* 351 was a potassium adduct molecular ion [(M+K)^+^]. As for fragment ions, *m*/*z* 150 and 133 were assigned to (aglycon)^+^ and (aglycon−OH)^+^, respectively. In the negative mode spectrum (Figure 3b), (M+Cl)^−^, (M−H)^−^, and (aglycon−H)^−^ ions were detected. In the cases of PA (Figure 3c,d), (M+K)^+^, (M)^+^, (M−OH)^+^, and (M−H)^−^ ions were detected. The ionization behaviors of PG and PA were similar to those of coniferyl and sinapyl monologs, as shown in Figures S4 and S5.

As for the coexisting inorganic species, K^+^ and Cl^−^ were widely detected in the phloem to xylem region in the transverse surfaces of frozen‐hydrated *P. thunbergii* wood (Figure S7). Moreover, their distribution areas were equal to or larger than that of phosphocholine ion that is the marker of plant biomembranes of phosphatidylcholine (Bollhöner et al., 2012; Khan & Williams, 1977; Li et al., 2006; Okumura et al., 2017). Therefore, the K^+^ or Cl^−^ adduct ions in the standard spectra (Figure 3; Figures S3, S4, and S5) could also be used for visualization when their intensities were significant enough.

The same *m*/*z* ion can arise from multiple compounds (Table 1). In such cases, the possibility of visualization should be carefully examined regarding their quantitative results, ionization efficiencies, and chemical conditions. Given the HPLC and IEC data (Figures 1 and 2), the secondary ions derived from saccharides should be stronger than those from MLGs in a high possibility. The contribution of MLs was smaller than that of MLGs and negligible in the differentiating xylem region. From these considerations and the resultant secondary ion distributions and intensities, several ion candidates were excluded from the imaging discussion; for example, *m*/*z* 149 (−) ion was overlapped with monosaccharide ions; *m*/*z* 163 (+) ion was overlapped with saccharide or water cluster‐derived ions; and *m*/*z* 381 ion (+) was overlapped with disaccharide or K‐adducted water cluster ions. Finally, the specific ions for cryo‐TOF‐SIMS visualization of MLGs were CF, *m*/*z* 180 (+) ion and PG, *m*/*z* 311 (−) ion.

### Cryo‐TOF‐SIMS imaging of MLGs

**Figure 4:**
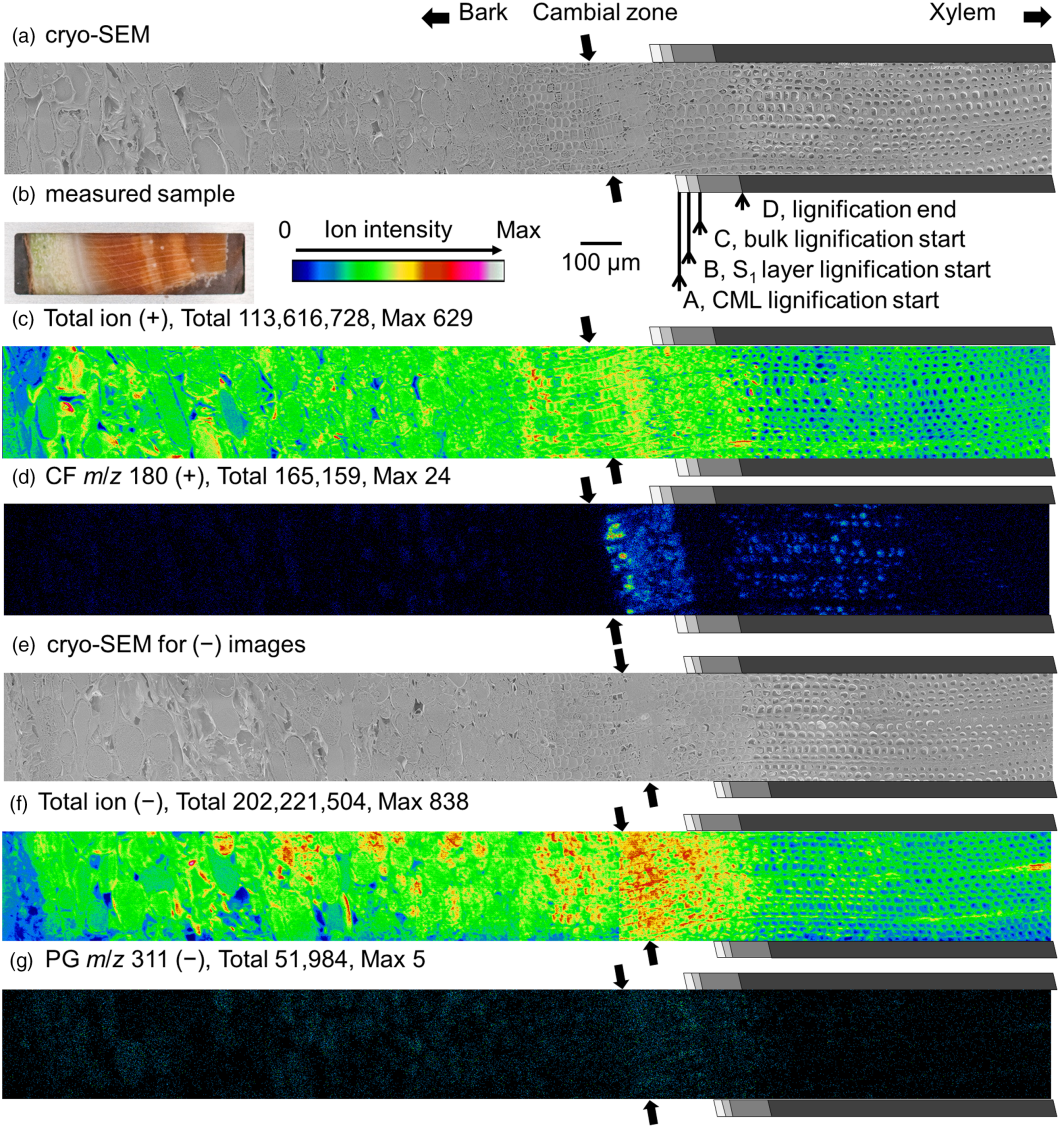
Cryo‐TOF‐SIMS/scanning electron microscopy (SEM) images of frozen‐hydrated transverse surfaces of compression wood. (a) Cryo‐SEM of the same region of (c). (b) The measured sample surface in positive ion mode. (c) Total ion (+). (d) CF *m*/*z* 180 (+) ion. (e) Cryo‐SEM of the same region of (f). (f) Total ion (−). (g) PG *m*/*z* 311 (−) ion. The gray‐scaled tetragons suggest the lignification stages determined by microscopic observations as follows: initiation of lignification of the compound middle lamella (CML) (A) outermost (S1) layer of secondary wall (B), and bulk (C). (D) Lignification end. Scale bar, 100 μm for SEM and SIMS images. Measurement in the negative ion mode (e, f, g) was conducted 3 days after measurement in the positive ion mode using the same block and a 100‐μm surface cutting for measuring the fresh surface.

**Figure 5:**
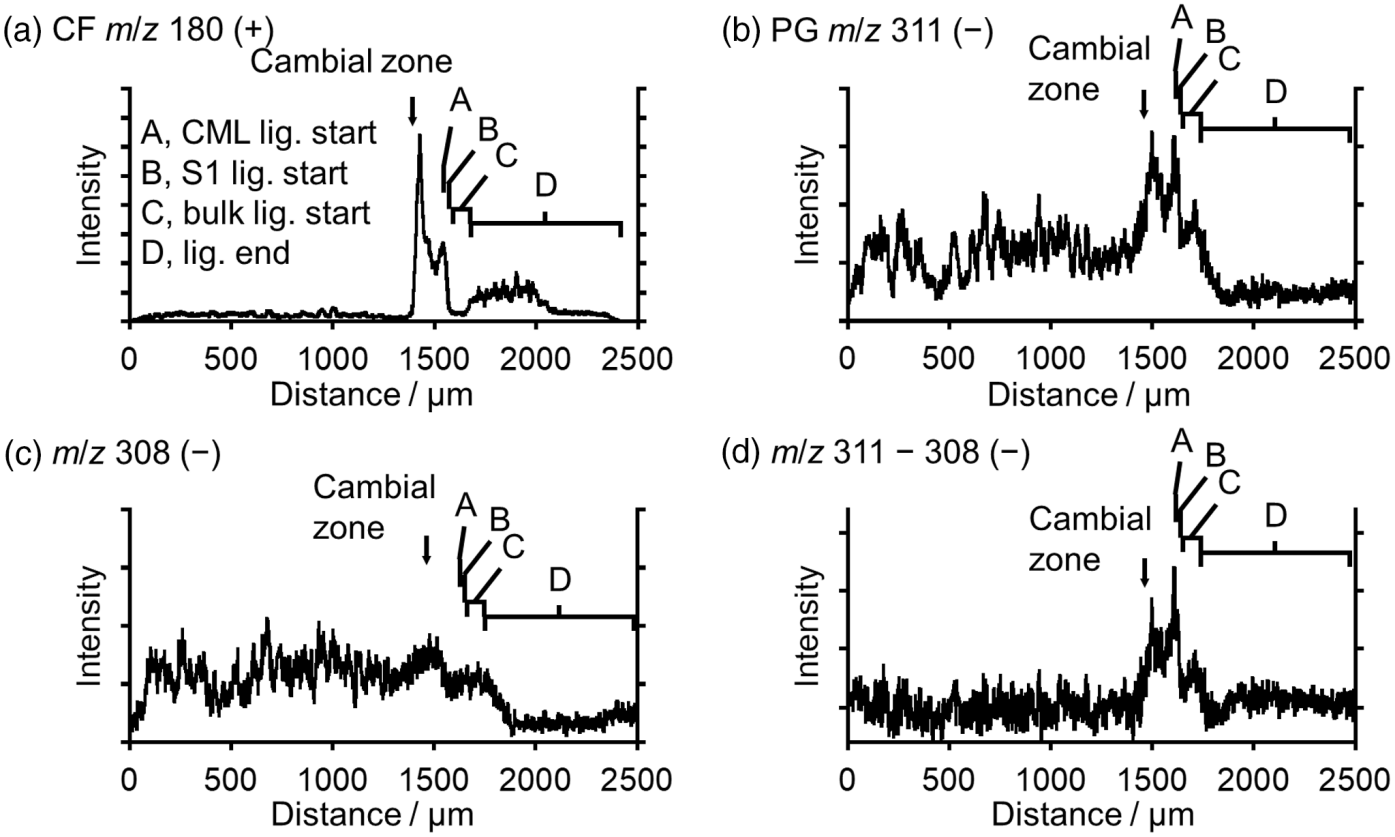
Radial distribution of (a) CF *m*/*z* 180 (+) ion, (b) PG *m*/*z* 311 (−) ion, and (c) *m*/*z* 308 ion counts. (d) The differential ion count profile between *m*/*z* 311 and 308 ions. Line profiles were obtained after image rotation of 12° for positive and 14° for negative images, shown in arbitrary unit (a.u.). The stages of cell wall lignification of the tracheid are suggested by initiation of lignification of the CML (A) outermost (S1) layer of secondary wall (B), and bulk (C). (D) Lignification end.

The cryo‐TOF‐SIMS ion images of compression wood are shown in Figure 4. The measured surfaces contained the phloem, cambial zone, and xylem regions (Figure 4b). Positive and negative images were obtained from the same sample block but on different surfaces. Radial distribution of *m*/*z* 180 (+) (CF) and 311 (−) (PG) ion counts are displayed in Figure 5 as line profiles after appropriate image rotation for adjusting the positions of the cambial zone and lignification stages (Figure S8). The position of the cambial zone and lignification stages of the tracheid were determined by microscopic observation using cryo‐SEM and visible, polarized, and UV light (Figure S9) and are shown in Figures 4 and 5 and Figure S8.

The distribution of CF (*m*/*z* 180 ion) showed apparent localization in the differentiating xylem region adjacent to the cambial zone (Figures 4d and 5a), which agrees with the HPLC results (Figure 1c). CF was significantly distributed from the cambial zone to the compound middle lamella (CML) and S_1_ layer lignification stages, and diminished rapidly around the start of bulk lignification. At the end of lignification, CF appeared slightly in the tracheid. Consistent with this result, our HPLC analysis also detected a small quantity of CF in the inner region where lignification was completed (Figure 1c). However, the ion intensity detected by cryo‐TOF‐SIMS was stronger than that expected from the HPLC results. One possible reason for this might be the ionization efficiency. In this region, the number of coexisting compounds in the tracheid sap may be less than that in the living tracheid. Ion suppression and competitive ionization are prevalent factors in mass spectrometry (Annesley, 2003). Further studies are required to improve quantification by cryo‐TOF‐SIMS imaging for comparing physiologically different tissues.

The count of the PG‐derived secondary ion (*m*/*z* 311) was low (Figure 4g). Supporting this result, HPLC determined that the actual amount of PG was one‐tenth of that of CF (Figure 1c). The *m*/*z* 311 ion showed maximum detection within the cambial zone to the differentiating xylem region (Figure 5b); however, the PG distribution was not consistent with the HPLC results (Figure 1c) in terms of the stronger signals detected in the phloem region than in the lignified xylem region. We suspect that this discrepancy may be owing to background noise in the phloem region. To confirm this, the *m*/*z* 308 ion count was recorded as a possible background ion species near the *m*/*z* 311 ion (Figure 5c). The *m*/*z* 308 ion count was higher in the phloem than in the lignified xylem. Based on this observation, we analyzed the differential ion count profile between *m*/*z* 311 and 308 ions (Figure 5d). As a consequence, the radial distribution of differential ion counts was overall consistent with the HPLC result of PG (Figure 1d), suggesting that it reflected the actual distribution of PG.

As for the other secondary ion candidates possibly derived from PG, the *m*/*z* 150 (+) ion was detected over the entire area of the measurement surface, and the distribution was inconsistent with the HPLC results. Therefore, the ion must be derived from plural compounds. The counts of *m*/*z* 351 (+) and 347 (−) ions were smaller than that of the *m*/*z* 311 ion, although their distributions were similar to that of the *m*/*z* 311 ion (Figure S10). Based on these observations, we deduced that the *m*/*z* 311 and *m*/*z* 180 ions help determine the *in planta* distribution of PG and CF, respectively (Figure 5a,d). Consequently, CF rapidly diminished, whereas PG remained slightly in stage C (Figure 5). The different behaviors of CF and PG are also shown in Figure 4(d,g). The *m*/*z* 311 ion was continuously detected after bulk lignification start, but the *m*/*z* 180 ion diminished in the region.

In opposite wood, only CF was detected using cryo‐TOF‐SIMS (Figure S11). CF was distributed from the cambial zone to the S_1_ lignification stage and rapidly diminished at the onset of lignification in the S_2_ and S_3_ layers. Although this behavior was similar to that observed in compression wood, CF (*m*/*z* 180 ion) was not detected after the end of lignification.

The distributions of endogenous CF and PG in *P. thunbergii* wood were successfully visualized using cryo‐TOF‐SIMS. Their distributions showed good agreement with the HPLC results and lignification stages. Along with the data from a previous study suggesting the assimilation of MLGs in these differentiating xylem regions (Fukushima & Terashima, 1991b: Fukushima et al., 1997), our data further support the view that these MLGs act as lignin precursors in compression and opposite woods of *P. thunbergii*. The distributions of CF and PG were similar; however, their apparent concentration patterns differed.

### Visualization of ML incorporation capacity using fluorescence‐tagged MLs

**Figure 6:**
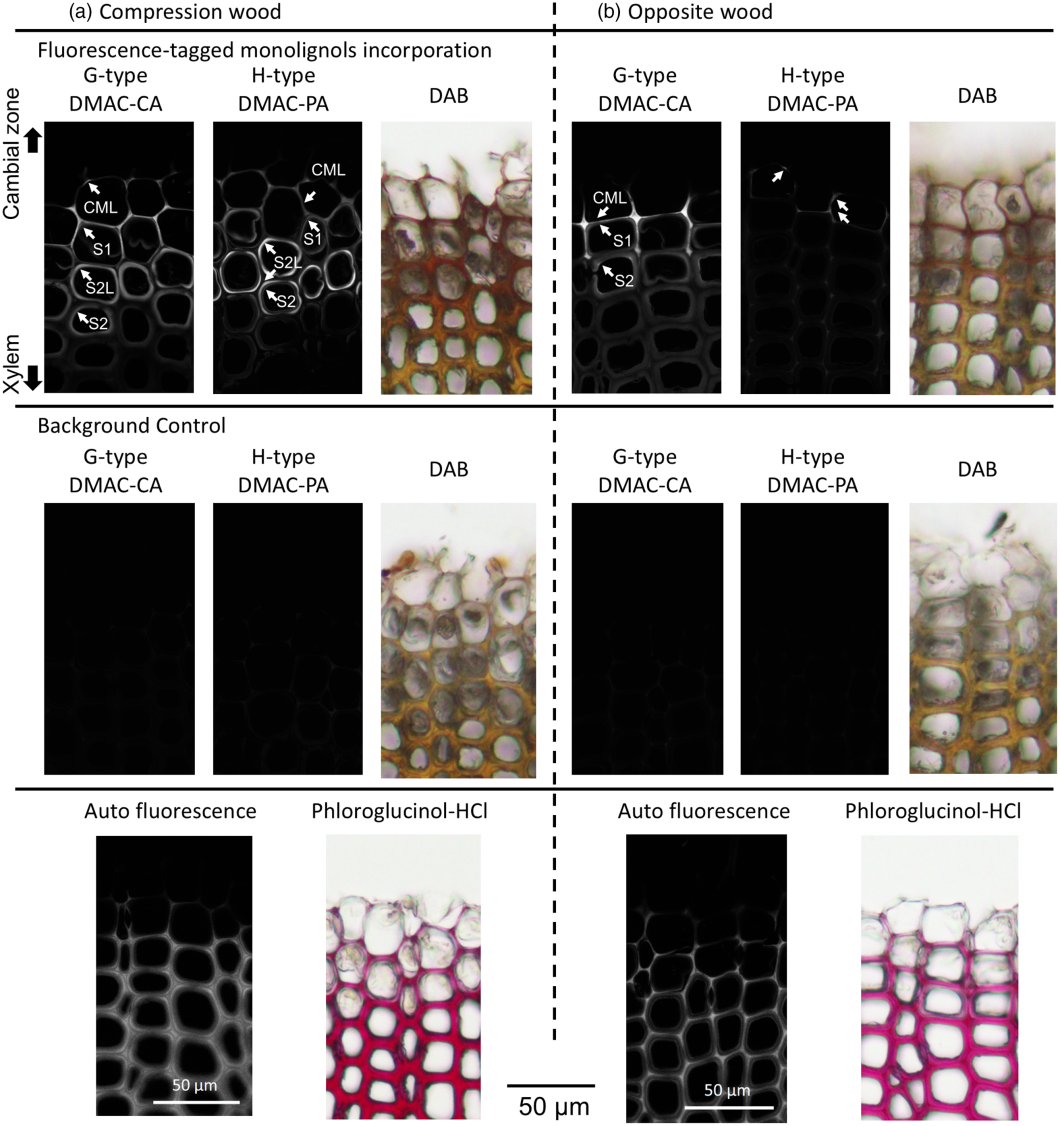
Oxidative enzyme‐mediated incorporation of fluorescence‐tagged monolignols (MLs) into the differentiating xylem region of (a) compression and (b) opposite woods. Images obtained for phenol oxidation activity, lignin deposition, and lignin autofluorescence are also summarized.

To further investigate the lignification processes utilizing different lignin precursors in compression and opposite woods of *P. thunbergii*, we performed cell wall imaging using fluorescence‐tagged MLs, which helps monitor active lignification sites through metabolic incorporation (Hiraide et al., 2021; Lee et al., 2019; Shuetz et al., 2014; Takenaka et al., 2018; Tobimatsu et al., 2013). A recent study has revealed distinct incorporation patterns of G and H unit‐type fluorescence‐tagged MLs in differentiating xylem tissues during the formation of normal and compression woods in *Chamaecyparis obtuse*, supporting the view that the spatial localization and biochemical characteristics of cell‐wall‐anchored ML‐oxidizing enzymes (e.g., laccases and peroxidases) impact spatial patterning of lignification in normal and compression woods of gymnosperms (Hiraide et al., 2021). In this study, we compared the oxidative enzyme‐dependent incorporation patterns of DMAC‐tagged G‐type (DMAC‐CA) and H‐type (DMAC‐PA) ML probes, which mimic canonical G‐type (CA) and H‐type (PA) MLs, respectively, between compression and opposite woods of *P. thunbergii* (Figure 6; Figure S12). Histochemical imaging of non‐labeled wood sections affirmed phenol oxidation activity and deposited lignin polymers in the samples (Figure 6).

Fluorescent tags were not detected in the fully lignified xylem cell walls (Figure 6). In a previous study, relatively low but detectable fluorescence has been observed in the fully lignified xylem cells (Hiraide et al., 2021). Therefore, we considered that the deposition of fluorescent tags was lower in the fully lignified xylem cell walls than in the differentiating xylem cell walls, probably owing to relatively low accessibility of the tags to the corresponding enzymes in the cell walls within a limited reaction time.

As shown in Figure 6, the G‐type DMAC‐CA and H‐type DMAC‐PA probes showed distinctively different incorporation patterns in compression and opposite wood sections of *P. thunbergii*. In compression and opposite wood sections labeled with DMAC‐CA, even DMAC fluorescence was detected in the CML and across the secondary cell wall layers, that is, S_1_, S_2_L, and S_2_ layers in compression wood (Figure 6a) and S_1_ and S_2_ layers in opposite wood (Figure 6b). This suggests that these lignification sites have comparable ML‐oxidizing enzyme activities for incorporating CA in G‐type lignin deposition. In contrast, in compression wood sections labeled with DMAC‐PA, strong DMAC fluorescence was explicitly detected in the S_2_L layer, with moderate signals in the CML, S_1_, and S2 layers. This result indicated that the compression‐wood‐specific S_2_L layer had considerably higher enzyme activity for incorporating PA in H‐type lignin deposition than those of the other lignification sites. However, in opposite wood sections labeled with DMAC‐PA, DMAC fluorescence was considerably lower than those observed in compression wood sections labeled with DMAC‐PA, and compression and opposite wood sections labeled with DMAC‐CA, suggesting a considerably weak enzyme activity for incorporating PA in H‐type lignin deposition in opposite wood. Overall, the different incorporation behaviors of the G‐type and H‐type fluorescence‐tagged MLs in compression and opposite woods of *P. thunbergii* are consistent with earlier observations in *C. obtuse* (Hiraide et al., 2021) and congruent with the distribution patterns of the G‐type and H‐type MLGs in *P. thunbergii* as revealed by cryo‐TOF‐SIMS and complementary HPLC analysis.

### Monomeric products of thioacidolysis

To analyze lignin composition in compression and opposite woods of *P. thunbergii*, we performed thioacidolysis, which selectively cleaves major β‐aryl ether‐linkages in the lignin polymer, followed by GC–MS analysis of lignin‐derived monomeric products. By coupling with pre‐methylation of free phenolic hydroxyl groups, thioacidolysis releases nonmethylated and methylated monomeric products derived from the internal units and end‐units, respectively, of the lignin polymer (Lapierre et al., 1988; Lapierre & Roland, 1988). Table 2 summarizes the results of the monomeric products of GT and HT released from the compression and opposite wood samples by thioacidolysis. The methylated products, GT‐OCH_3_ and HT‐OCH_3_, are thought to be derived from the G and H units of the phenolic end‐groups of the lignin polymer chains. The GT unit was the primary product in both samples, whereas the HT unit was below the detection limit in the opposite wood sample. Methylated monomeric thioacidolysis products were obtained by methylation. The resultant ‐OCH_3_ ratio suggested the end‐group ratio of the unit structure. The ‐OCH_3_ ratio of the GT unit in compression and opposite wood samples was 7–11%. In contrast, the ratio of the HT unit in compression wood samples was significantly high (84%). Collectively, these results confirmed the compression‐wood‐specific deposition of H‐type lignin and preferential incorporation of H‐units into the end‐units of lignin polymers. A similar result has been previously reported for compression wood of *Pinus radiate* (Lapierre et al., 1988; Lapierre & Roland, 1988).

**Table 2:** Yields of monomeric products of thioacidolysis

Samples	Yield ± S.D. (μmol/sample‐g)				‐OCH_3_ ratio (%)	
	GT	GT‐OCH_3_	HT	HT‐OCH_3_	GT	HT
Compression wood	314 ± 148	n.d.	43 ± 5	n.d.	‐	‐
Opposite wood	371 ± 14	n.d.	n.d.	n.d.	‐	‐
Compression wood (methylated)	281 ± 56	23 ± 3	8 ± 3	43 ± 7	7	84
Opposite wood (methylated)	223 ± 43	29 ± 7	n.d.	n.d.	11	‐

n.d., not detected; S.D., standard deviation (*n* = 3).

### Difference between the G and H units

CF and PG assimilation into lignin in compression and opposite woods of *P. thunbergii* has been previously demonstrated by autoradiography experiments using isotopically labeled CF and PG (Fukushima & Terashima, 1991b; Terashima & Fukushima, 1988). In compression wood, the assimilation of administered PG into lignin in the cell wall starts simultaneously as CML lignification starts. The assimilation of PG continues, and the maximum assimilation occurs in the first half of the S_2_ layer forming period. The assimilation of CF also starts at the onset of CML lignification; however, assimilation decreases once and increases in the latter half of cell wall lignification. The maximum CF assimilation agrees with that of the bulk lignification period. Considering these previous results and the results obtained in this study, we aimed to discuss and arrange the differences between G and H units in terms of precursor storage, enzymatic selectivity, and their structure in lignin of compression and opposite woods. In the following description, [adm], [fluo], and [endo] refer to the results observed in MLG autoradiography experiments (Fukushima & Terashima, 1991b; Terashima & Fukushima, 1988), cell wall imaging using fluorescence‐tagged monolignols (this study), and endogenous MLG distributions as revealed by cryo‐TOF‐SIMS and HPLC (this study), respectively.

Lignification starts in the CML region. CF·PG [adm] and CA·PA [fluo] can be assimilated into CML lignin, and CF and PG are stored in the tracheid in this region [endo]. Subsequently, CF assimilation decreases once and increases to the maximum level in the bulk lignification stage [adm]. During bulk lignification, the apparent storage of CF diminishes [endo]. Here, the distribution visualized by cryo‐TOF‐SIMS suggests the apparent storage amount and not the biosynthetic activity; therefore, the decrease in CF storage in the bulk lignification stage is reasonable if the consumption speed is equal to or higher than the biosynthetic speed in this region.

**Figure 7:**
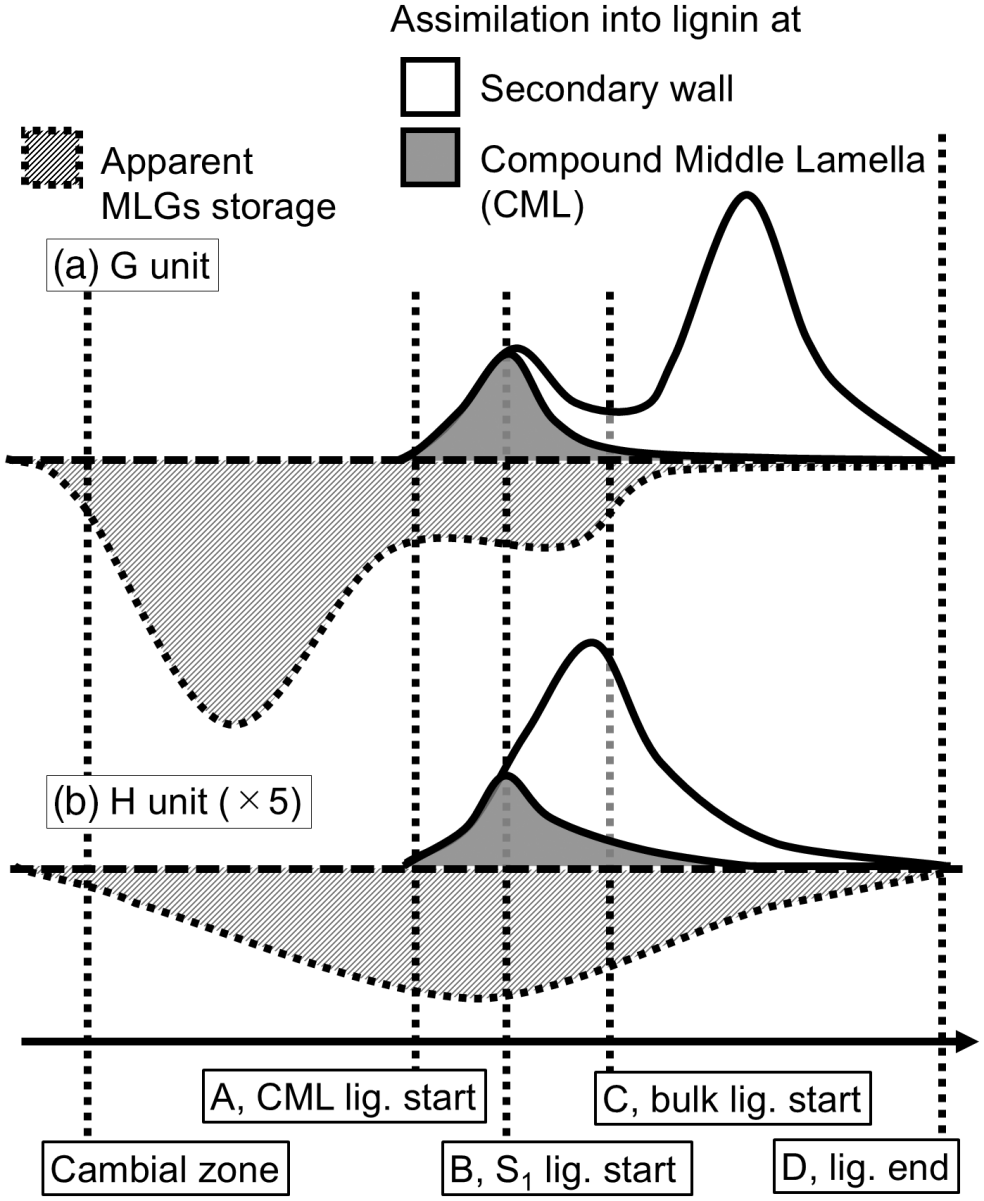
Schematic monolignol glucosides (MLG) distribution in compression wood as shown in this study. Their deposition into lignin [rearranged from Fukushima and Terashima (1991b)] and apparent precursor storages for the (a) G and (b) H units. The lignification (lig.) stages of the cell wall of the tracheid are presented by A, B, C, and D in the same manner in Figures 4 and 5.

In contrast, PG is gradually assimilated into lignin [adm], specifically in the S_2_L region [fluo]. PG is stored in the tracheid between the lignification periods, and its decrease with the start of bulk lignification is relatively slower than that of CF [endo]. This may explain why the H‐unit is mainly detected as the end group. In opposite wood, PG is stored in little amount [endo] and can be assimilated into lignin around the CML region [fluo]. These behavioral differences between the G and H precursors suggest the possibility of individual mechanisms regulating the heterogeneous G‐ and H‐unit structures of lignin. These processes for compression wood are schematically illustrated in Figure 7. In the opposite wood region, only the G unit (Figure 7) is applicable. In Figure 7, the heights of storage and assimilation are proportional to the HPLC and thioacidolysis results, respectively, and those of the H unit are multiplied by 5; however, the scale is not quantitative, and this is only a conceptual diagram.

Thioacidolysis selectively cleaves β‐aryl ether‐linkages. Therefore, the resultant G and H unit‐derived product yields were not the same as their total amount in lignin. Nevertheless, the end‐group ratio, as summarized in Table 2, was relatively high in the H unit, suggesting some difference between the G and H units in lignin. Previous studies on lignin structure have suggested that the abundance of β‐aryl ether‐linkages is apparently insensitive to the increment of H units in softwood compression woods (Hirayama et al., 2019; Saito & Fukushima, 2005), although genetic manipulations to increase H lignin units in transgenic plants generally deplete β‐aryl ether‐linkages in the resultant lignin polymers (Bonawitz et al., 2014; Ralph et al., 2006; Takeda et al., 2018). The high end‐group ratio of H lignin determined in this study might be at least partially owing to the relatively lower molecular mass of H‐rich lignin than that of typical G/S lignin (Bonawitz et al., 2014).

Additionally, the H unit was not detected in opposite wood despite the possibility of PG detection and assimilation. The amount of PG in the differentiating xylem region of opposite wood was one‐fifth of that in compression wood; therefore, the H unit should have been detectable if the lignin structure (i.e., bonding pattern frequencies) was same in compression and opposite woods except for the unit ratio. One possible hypothesis is that PG is used as a precursor of CF and is not incorporated into lignin as an H‐unit. The transformation of administered PG into the G‐units of lignin has been previously reported (Terashima & Fukushima, 1988). A detailed discussion is necessary considering the specific transport mechanisms for CF (Tsuyama et al., 2013) and PG (Tsuyama et al., 2019) and tissue‐specific localization of oxidative enzymes for lignification (Chou et al., 2018; Hiraide et al., 2021; Hoffmann et al., 2020; Shuetz et al., 2014; Wang et al., 2020).

The condensed‐type structures may explain another hypothesis. A previous study (Fukushima & Terashima, 1991b; Terashima & Fukushima, 1988) has indicated that lignin in the CML is rich in condensed‐type linkages compared to that in lignin of the secondary wall. If PG in opposite wood was consumed during CML lignification apart from β‐aryl ether‐linkages and not synthesized and used as a lignin precursor after the period, the H unit might not be incorporated in secondary wall lignin and could not be detected by thioacidolysis. However, excess PG in compression wood, which is gradually consumed during the lignification period, can be assimilated into lignin of the secondary wall and detected by thioacidolysis. Quantitative discussions regarding lignin in the CML and condensed‐type H units are essential for clarifying the remaining topics.

## MATERIAL AND METHODS

### Materials

*P. thunbergii* seedlings (3‐year‐old) were grown in 30° tilted condition from February 28, 2017. The inclined *P. thunbergii* samples were cut and divided into blocks of compression and opposite wood and cut further into small blocks (central angle, *π*/16 and axial length, 20 mm) containing bark, cambial zone, and xylem on July 3, 2017. The small blocks were quickly frozen at −160°C using liquid Freon 22 (Du Pont de Nemours, Inc., Wilmington, DE, USA) and stored at −80°C before use. The cultivation conditions, and resultant compression and opposite woods are shown in Figure S1. The seedlings were subjected to chemical analyses, microscopic observations, and imaging mass spectrometry. Although preliminary experimental data were confirmed using several individuals, the whole data in this report were from one individual sample to unite this chemical information with detailed microscopic observations.

Chemical reagents were purchased from Kishida Chemical Co., Ltd. (Osaka, Japan), Kanto Chemical Co., Inc. (Tokyo, Japan), FUJIFILM Wako Pure Chemical Corporation (Osaka, Japan), Tokyo Chemical Industry Co., Ltd. (Tokyo, Japan), and Merck KGaA (Darmstadt, Germany). CA, PA, CF, and PG were synthesized as previously described (Terashima et al., 1996). Dimethylaminocoumarin (DMAC)‐tagged CA and PA were synthesized as previously described (Tobimatsu et al., 2011, 2013).

### HPLC and ion exchange chromatography (IEC) analyses

Frozen samples were cut successively to tangential sections of 50‐μm thickness from the bark to the xylem using a sliding microtome (REM‐710; Yamato Kohki Industrial Co. Ltd., Saitama, Japan) equipped with a cryo sample stage (MC‐802A). Prior to tangential sectioning, the debarked xylem from each block was cut and removed (Figure S1). Three sections were packed in a microtube and extracted using 1 mL water for 30 min at 100°C and 150 min at 25°C. Here, section number 1 means the sum of three section and corresponds to 150‐μm thickness. The extract was filtered, frozen, freeze‐dried, and dissolved in 0.1 mL water. Individual sample sets of three blocks were used for the HPLC and IEC measurements, and the average values and standard deviations were estimated. The residual sections in microtubes were freeze‐dried and weighed to determine the position of the cambial zone containing the lowest‐weight section (Figure S2).

The amounts of CA, PA, CF, and PG were quantified by HPLC. The measuring condition was as follows: apparatus, two LC‐6ADs/DGU‐12A/SIL‐20 AC/CBM‐20A/CTO‐10A/SPD‐10A (Shimadzu corporation, Kyoto, Japan); column, TSK‐GEL ODS‐100A (4.6 mm ID × 200 mm L; Tosoh Corporation, Tokyo, Japan); temperature, 40°C; flow rate, 1 mL min^−1^; eluent, H_2_O (solvent A) and a methanol to acetonitrile ratio of 6:1 (v/v, solvent B) with gradients of B 10% for 10 min, B 10–20% for 10 min, B 20–60% for 10 min, and B 60–10% for 15 min.

Monosaccharides and disaccharides were quantified by IEC with the following measuring condition: apparatus, DIONEX ICS‐3000 (Thermo Fisher Scientific K.K., Tokyo, Japan); column, CarboPac PA‐1 (2.0 mm ID × 250 mm L); temperature, 30°C; flow rate, 0.3 mL min^−1^; eluent, H_2_O (solvent A), 100 mM aqueous sodium hydroxide aqueous solution (solvent B), and 100 mM NaOH, and 1 M aqueous sodium acetate. (solvent C) with gradients of B 0.5% and C 0% for 45 min, B 0% and C 100% for 10 min, B 100% and C 0% for 10 min, and B 0.5% and C 0% for 15 min for galactose, mannose, and xylose, or gradients of B 50% and C 0% for 50 min, B 0% and C 100% for 10 min, B 100% and C 0% 10 for min, and B 50% and C 0% for 15 min for sucrose, fructose, glucose, and arabinose.

The *t*‐test was conducted using the data analysis add‐in tool in Microsoft 365 Excel (version 2408). Within the selected sections, the *p* value was evaluated using the TTEST function with two‐tailed and equal variance.

### Cryo‐TOF‐SIMS/SEM analyses

Frozen samples were fixed in a sample holder by ice embedding. Clean and flat sample surfaces were prepared using a sliding microtome in a glove box under a dry N_2_ atmosphere (<−10°C). The samples were transferred using a cryo‐vacuum shuttle system (Kuroda et al., 2013; Masumi et al., 2014), and secondary ion images were obtained using cryo‐TOF‐SIMS. The measuring condition was as follows: apparatus, TRIFT III spectrometer (ULVAC PHI Inc., Chigasaki, Japan); primary ion, 22 keV Au_3_^+^ at current 5–7 nA; raster size, 300 × 300 μm^2^ (256 × 256 pixels); pulse width, 13 ns (non‐bunched for image) or 1.8 ns (bunched for spectrum); mass range, *m*/*z* 0.5–1850; spot size, 1.0 μm (non‐bunched for image); degree of vacuum, <10^−6^ Pa; sample temperature, −130 to−120°C; a low energy pulsed electron gun for surface charge compensation, 30.0 eV; and measurement time, 30 min for each image. Standard chemicals (10 mM) and KCl (10 mM) were dissolved to prepare standard solutions, frozen, and measured using the procedure same as in the bunched mode. The calibration curves used were as follows: positive mode; (CH)^+^ 13.0078, [(H_2_O)_2_+H]^+^ 37.0289, [(H_2_O)_4_+H]^+^ 73.0500, and [(H_2_O)_11_+H]^+^ 199.1238; negative mode; (OH)^−^ 17.0027, [(H_2_O)_3_−H]^−^ 53.0238, [(H_2_O)_4_−H]^−^ 71.0343, and [(H_2_O)_11_−H]^−^ 197.1081.

After cryo‐TOF‐SIMS analysis, the samples were transferred to cryo‐SEM (S‐3400N; Hitachi High‐Technologies Corporation) equipped with cryo sample stage C1001 (Gatan, Inc., CA, USA) by a cryo‐vacuum shuttle system, and the same surface region was observed after an appropriate freeze etching process at −90°C. The observation conditions were as follows: acceleration voltage, 1.5 kV; temperature, −120°C; and degree of vacuum, <10^−3^ Pa. Continuous SEM images were obtained using Photoshop CS5 Extended (Adobe Inc., San Jose, CA, USA).

All cryo‐TOF‐SIMS data were obtained as a raw data file containing a full mass spectrum at every 256 × 256 pixel. The obtained images were connected using WinCadence v.5.1.2.8 (ULVAC‐PHI Incorporation) and MatLab R2019a (The MathWorks, Inc., Natick, MA, USA) with PLS Toolbox v.8.8.1 (Eigenvector Research, Inc., Manson, WA, USA) without any ion count normalization. The color scale for the obtained united image was changed using ImageJ (Rasband, 1997). The National Institutes of Health, Bethesda, ML, USA). The rotated images and their line profiles were obtained using ImageJ.

### Other microscopic analyses

The samples were divided into small blocks (axial 2 mm × tangential 1 mm × radial 1 mm) and quickly frozen. The frozen blocks were applied to freeze‐substitution in glutaraldehyde‐acetone solution for 3 days at −80°C, 2 days at −30°C, and 1 day at 4°C, and then stored at room temperature. The block was embedded in epoxy resin and cut into 2, 1, or 0.25‐μm thick sections by a motorized rotary microtome (Microm HM350; Thermo Fisher Scientific K.K.). The sections of 2‐μm thickness were stained with toluidine blue and observed using an optical microscope (BX50; Olympus Corporation, Tokyo, Japan). The sections of 1‐μm thickness were observed using a polarized optical microscope (BX50). The UV photographs of 0.25‐μm thick sections were captured using a UV microspectrophotometer (MPM800, Carl Zeiss AG, Germany) at 280 nm wavelength. Continuous images were prepared using Photoshop CS5 Extended.

Cell wall imaging using fluorescence‐tagged MLs and histochemical methods were performed as previously described (Hiraide et al., 2021). For imaging with fluorescence‐tagged MLs, serial transverse sections (60‐μm thickness) were freshly cut from compression and opposite wood samples (stored at −80°C after harvest) using a sliding microtome, washed in sodium acetate buffer (20 mM, pH 5.0), and then treated with 10 μM DMAC‐tagged CA or PA in sodium acetate buffer supplemented with exogenous H_2_O_2_ (0.5 mM) for 2 h at room temperature. The labeled sections were thoroughly washed with ethanol to remove nonincorporated fluorescence‐tagged ML probes. The incorporated fluorescence tags were visualized using a Leica TCS SPE confocal laser microscope (CLSM) (Leica Microsystems, Wetzlar, Germany) with excitation/emission wavelengths at 405/440–480 nm. To obtain the background control sections, heat treatment at 85°C for 1 h was applied to the sections before they were subjected to the same labeling treatments and CLSM imaging under the same laser strength and emission gain. Lignin autofluorescence images of nonlabeled control sections were obtained at excitation/emission wavelengths of 405/415–600 nm. Histochemical imaging using 3,3′‐diaminobenzidine (DAB) for detecting phenol oxidation activity and using phloroglucinol‐HCl for detecting deposited lignin was conducted as previously described (Hiraide et al., 2016, 2021).

### Methylation–thioacidolysis analysis

The current‐year xylem of the small sample blocks was cut into tangential sections to obtain compression and opposite wood samples for methylation and thioacidolysis reactions. The sections were pre‐extracted with hot water (8 h) and then with acetone (8 h) in a Soxhlet extractor, cut into 5 × 5‐mm^2^ pieces, and permethylated with diazomethane in dehydrated dioxane (Lapierre et al., 1988; Lapierre & Roland, 1988). The methylated and nonmethylated sections were subjected to thioacidolysis (Lapierre et al., 1985; Nimz, 1974). The approximate sample weight for each reaction was 2 mg. The resultant lignin‐derived monomeric products were silylated and injected into a gas chromatography–mass spectrometry (GC–MS) column under the following condition: apparatus, TRACE 1300GC/ITQ 900 (Thermo Fisher Scientific K.K.); column, Rxi‐1 ms (30 m × 0.32 mm I.D., 0.25 μm film thickness); temperature program, 180°C for 1 min, 180–230°C for 25 min, 230–300°C for 4 min 20 s, and 300°C for 5 min; column flow, 15.0 mL min^−1^; carrier gas, He; and injection temperature, 250°C. Monomeric products were detected and quantified using GC–MS as previously described (Lapierre et al., 1991; Roland et al., 1992). Reactions and measurements were performed in triplicates for each sample.

To determine the response factors, thioacidolysis monomer products were synthesized as previously reported (Matsushita et al., 2020; Shimizu et al., 2021), methylated with diazomethane, and analyzed by GC–MS as described above. The response factors for the G‐unit‐derived thioacidolysis degradation products (GT), methylated GT (GT‐OCH_3_), H‐unit‐derived thioacidolysis degradation products (HT), and methylated HT (HT‐OCH_3_) were 0.386, 0.406, 0.414, and 0.494, respectively.

## AUTHOR CONTRIBUTIONS

NM, DA, YM, and FK conceived the research. NM, DA, SF performed TOF‐SIMS experiments. YM and HM synthesized standard materials. NM, DA, MY, HH, and YT carried out the microscopic observations. HH and YT conducted fluorescence‐tag experiments. NM and DA wrote the manuscript. YM, YT, and FK edited the manuscript. All authors read and contributed to the manuscript.

## CONFLICT OF INTEREST

The authors declare that they have no competing interests.

## Supporting information


**Table S1.** Cryo‐TOF‐SIMS ions for MLs, MLGs, and saccharides.
**Figure S1.** Cultivation of the compression and opposite woods of *Pinus thunbergii*.
**Figure S2.** Dry weight variation of the serial tangential sections.
**Figure S3.** Radial distribution of saccharides.
**Figure S4.** Cryo‐TOF‐SIMS spectra of MLs.
**Figure S5.** Cryo‐TOF‐SIMS spectra of MLGs.
**Figure S6.** Cryo‐TOF‐SIMS spectra of saccharides.
**Figure S7.** Cryo‐TOF‐SIMS/SEM images of freeze‐fixed *Pinus thunbergii* compression wood.
**Figure S8.** Rotated cryo‐TOF‐SIMS images.
**Figure S9.** Optical, polarized optical, and UV microscopy images.
**Figure S10.** Radial distribution of cryo‐TOF‐SIMS positive and negative ions.
**Figure S11.** Cryo‐TOF‐SIMS/SEM images of freeze‐fixed *Pinus thunbergii* opposite wood.
**Figure S12.** Oxidative enzyme‐mediated incorporation of fluorescence‐tagged monolignols.

## Data Availability

The data that support the findings of this study are available from the corresponding author upon reasonable request.
