# Supplementary material for: The distribution of monolignol glucosides coincides with lignification during the formation of compression wood in Pinus thunbergii
Source: Plant J. 2024 Dec 14;121(2):e17209. doi: 10.1111/tpj.17209 (PMC11776043; doi:10.1111/tpj.17209)
Supplement: Supplementary file 1 — Table S1. Cryo‐TOF‐SIMS ions for MLs, MLGs, and saccharides. Figure S1. Cultivation of the compression and opposite woods of Pinus thunbergii. Figure S2. Dry weight variation of the serial tangential sections. Figure S3. Radial distribution of saccharides. Figure S4. Cryo‐TOF‐SIMS spectra of MLs. Figure S5. Cryo‐TOF‐SIMS spectra of MLGs. Figure S6. Cryo‐TOF‐SIMS spectra of saccharides. Figure S7. Cryo‐TOF‐SIMS/SEM images of freeze‐fixed Pinus thunbergii compression wood. Figure S8. Rotated cryo‐TOF‐SIMS images. Figure S9. Optical, polarized optical, and UV microscopy images. Figure S10. Radial distribution of cryo‐TOF‐SIMS positive and negative ions. Figure S11. Cryo‐TOF‐SIMS/SEM images of freeze‐fixed Pinus thunbergii opposite wood. Figure S12. Oxidative enzyme‐mediated incorporation of fluorescence‐tagged monolignols. [file TPJ-121-0-s001.pdf]

Supporting Information

The distribution of monolignol glucosides coincides with lignification during the formation of compression wood in *Pinus thunbergii*

MAEDA Naoki[1], AOKI Dan[1*], FUJIYASU Syunya[1], MATSUSHITA Yasuyuki[2], YOSHIDA Masato[1], HIRAIDE Hideto[3,4], MITSUDA Hayato[1], TOBIMATSU Yuki[4], and FUKUSHIMA Kazuhiko[1]

[1] Graduate School of Bioagricultural Sciences, Nagoya University, Nagoya 464-8601, Japan
[2] Institute of Agriculture, Tokyo University of Agriculture and Technology, Tokyo 183-8509, Japan
[3] Graduate School of Agriculture, Kyoto University, Kitashirakawa-oiwakecho, Kyoto, 606-8502, Japan
[4] Research Institute for Sustainable Humanosphere, Kyoto University, Gokasho, Uji 611-0011, Japan
*To whom correspondence: aoki.dan@nagoya-u.jp

Contents

Table S1 Cryo-TOF-SIMS Ions for MLs, MLGs, and Saccharides.
Fig. S1 Cultivation of the compression and opposite woods of *P. thunbergii*.
Fig. S2 Dry weight variation of the serial tangential sections.
Fig. S3 Radial distribution of saccharides.
Fig. S4 Cryo-TOF-SIMS spectra of MLs.
Fig. S5 Cryo-TOF-SIMS spectra of MLGs.
Fig. S6 Cryo-TOF-SIMS spectra of saccharides.
Fig. S7 Cryo-TOF-SIMS/SEM images of freeze-fixed *P. thunbergii* compression wood.
Fig. S8 Rotated cryo-TOF-SIMS images.
Fig. S9 Optical, polarized optical, and UV microscopy images.
Fig. S10 Radial distribution of cryo-TOF-SIMS positive and negative ions.
Fig. S11 Cryo-TOF-SIMS/SEM images of freeze-fixed *P. thunbergii* opposite wood.
Fig. S12 Oxidative enzyme-mediated incorporation of fluorescence-tagged monolignols.

Table S1 Cryo-TOF-SIMS Major Ions and Chemical Structures for (a) MLs, (b) MLGs, and Saccharides.

	m/z	
	Positive mode	Negative mode
PA	$[M-OH]^+$ 133, $[M]^+$ 150, $[M+K]^+$ 189	$[M-H]^-$ 149
CA	$[M-OH]^+$ 163, $[M]^+$ 180, $[M+K]^+$ 219	$[M-H]^-$ 179
Sinapyl alcohol	$[M-OH]^+$ 193, $[M]^+$ 210, $[M+K]^+$ 249	$[M-H]^-$ 209
PG	$[aglycon]^+$ 150, $[M+K]^+$ 351	$[aglycon-H]^-$ 149, $[M-H]^-$ 311, $[M+Cl]^-$ 347
CF	$[aglycon]^+$ 180, $[M+K]^+$ 381	$[aglycon-H]^-$ 179, $[M-H]^-$ 341, $[M+Cl]^-$ 377
Syringin	$[aglycon]^+$ 210, $[M+K]^+$ 411	$[aglycon-H]^-$ 209, $[M-H]^-$ 371, $[M+Cl]^-$ 407
pentose	$[M+K]^+$ 189	$[M-H]^-$ 149, $[M+Cl]^-$ 185
hexose	$[M+K]^+$ 219	$[M-H]^-$ 179, $[M+Cl]^-$ 215
sucrose	$[M+K]^+$ 381	$[M-H]^-$ 341, $[M+Cl]^-$ 377

(a)

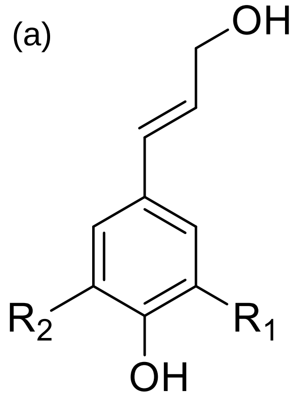

(b)

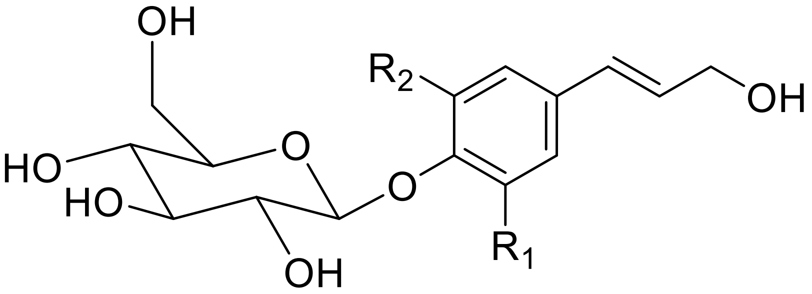

PA: $R_1 = R_2 = H$
CA: $R_1 = OCH_3$, $R_2 = H$
Sinapyl alcohol: $R_1 = R_2 = OCH_3$

PG: $R_1 = R_2 = H$
CF: $R_1 = OCH_3$, $R_2 = H$
Syringin: $R_1 = R_2 = OCH_3$

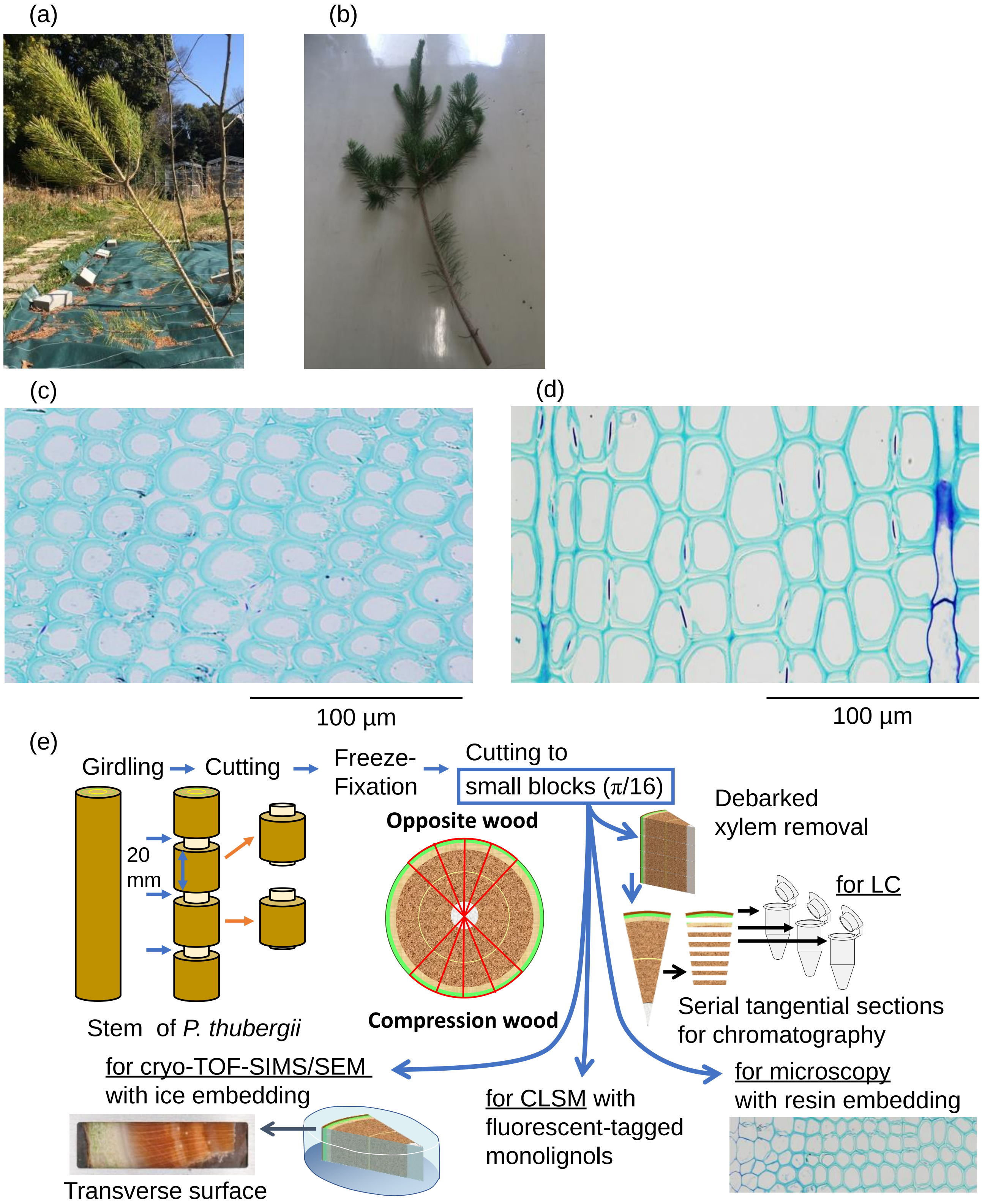

Fig. S1 Cultivation of the compression and opposite woods of *P. thunbergii*. Sample photographs at (a) just after the tilting and (b) the sampling date. Microscopic observations confirmed the resultant compression and opposite wood formation. Microscopy images of toluidine blue stained (c) compression and (d) opposite woods. Scale bars are 100 µm for (c) and (d). (e) A small block sample preparation scheme for LC, microscopies, and cryo-TOF-SIMS/SEM.

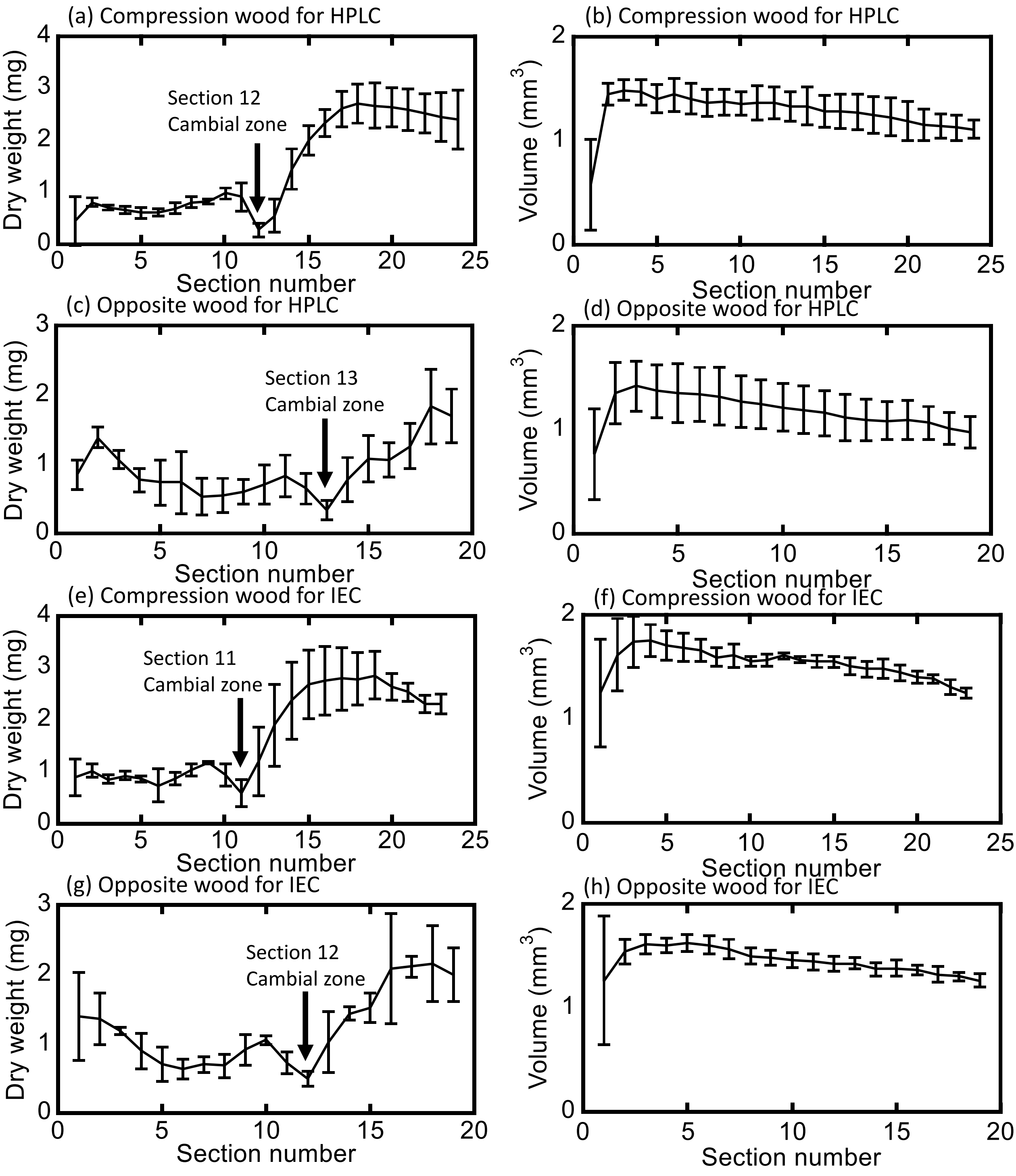

Fig. S2 Results for (a,c,e,g) dry weight and (b,d,f,h) volume variation of the serial tangential sections for (a,b,e,f) compression and (c,d,g,h) opposite wood samples used for chromatography measurements. The average and standard deviations for each section were obtained using 3 different sample blocks. The volumes of sections were evaluated using 3 sections of 50-μm thickness. A set of three sections were put into the same microtube and named as one section with a 150-μm thickness. The arrows in (a,c,e,g) mean the cambial zone containing section, as determined by the lightest weight before the weight increment in differentiating xylem region.

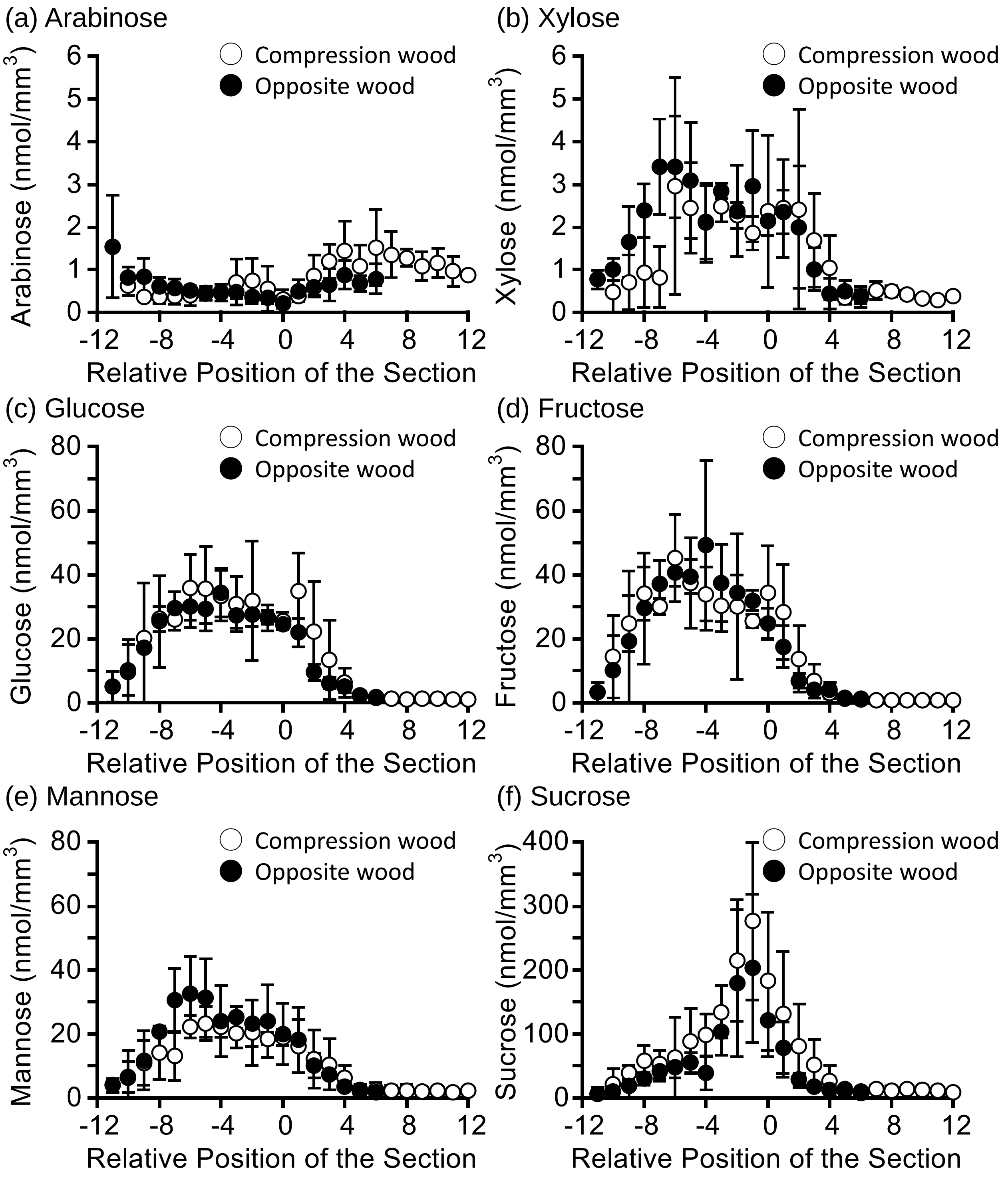

Fig. S3 The radial distribution of saccharides in compression and opposite woods of *P. thunbergii*, evaluated by IC. Results for (a) Ara, (b) Xyl, (c) Glc, (d) Fru, (e) Man, and (f) Suc. The numbers of the cambial zone containing sections were determined by the dry weight variation of the sections, as shown in Fig. S2, and the relative position of the cambial zone containing section was fixed as 0. Relative position of the section corresponds to phloem region (minus) and xylem region (plus). The means and standard deviations for each section were obtained from three sets of measurements using individual sample blocks cut from the same disk.

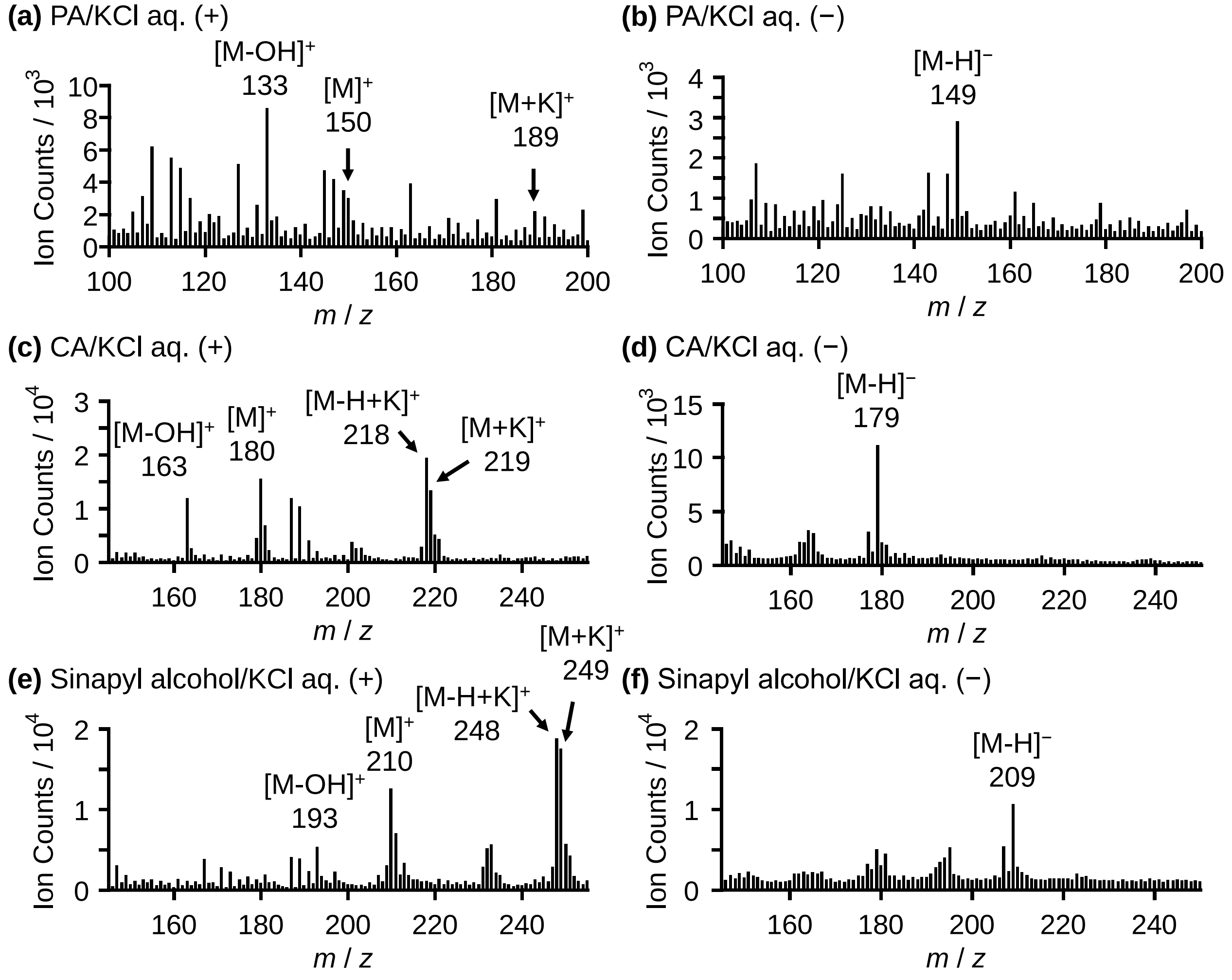

Fig. S4 Cryo-TOF-SIMS spectra of MLs dissolved in KCl aq. in positive and negative modes.

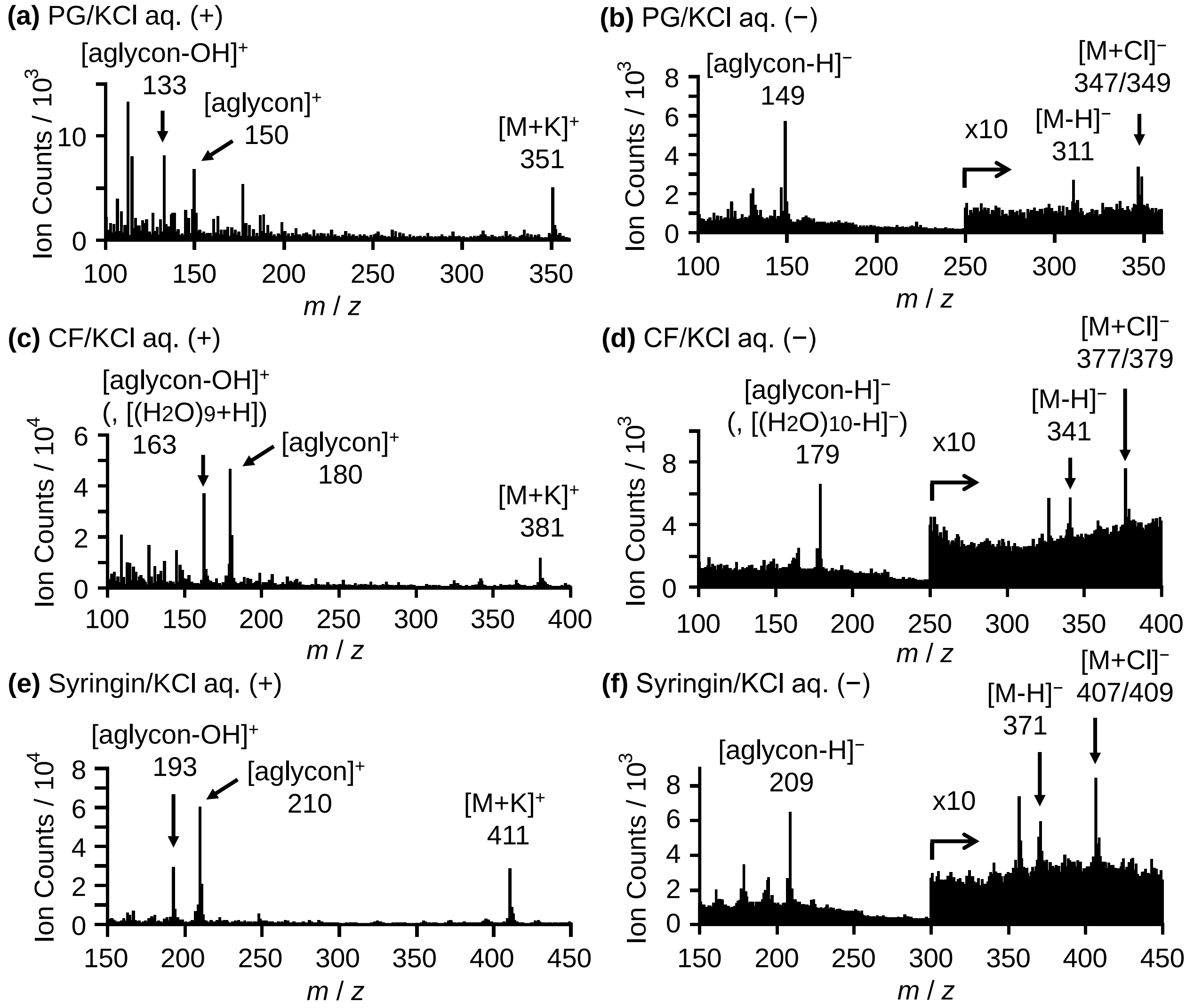

Fig. S5 Cryo-TOF-SIMS spectra of MLGs dissolved in KCl aq. in positive and negative modes.

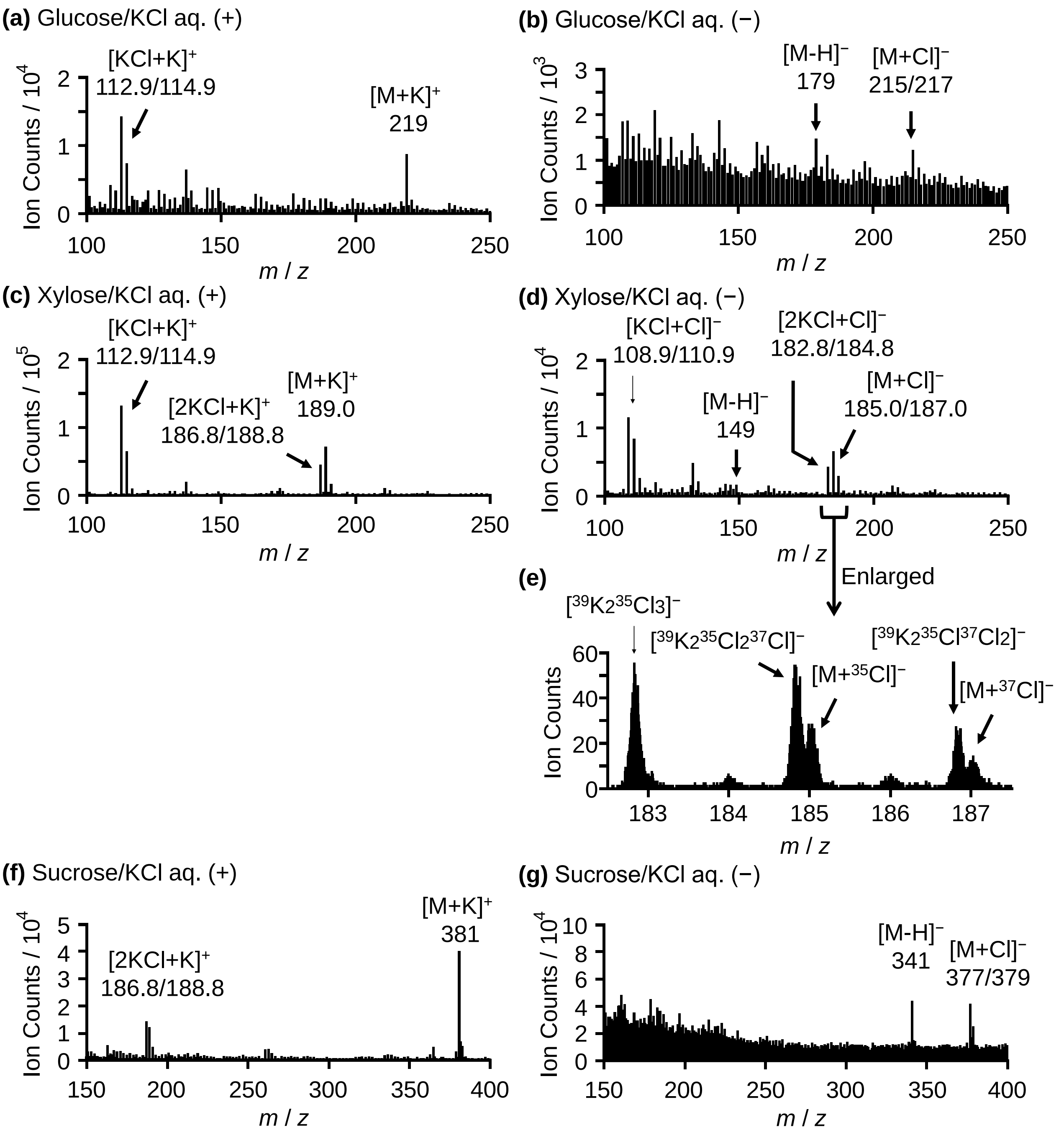

Fig. S6 Cryo-TOF-SIMS spectra of saccharides dissolved in KCl aq. in positive and negative modes.

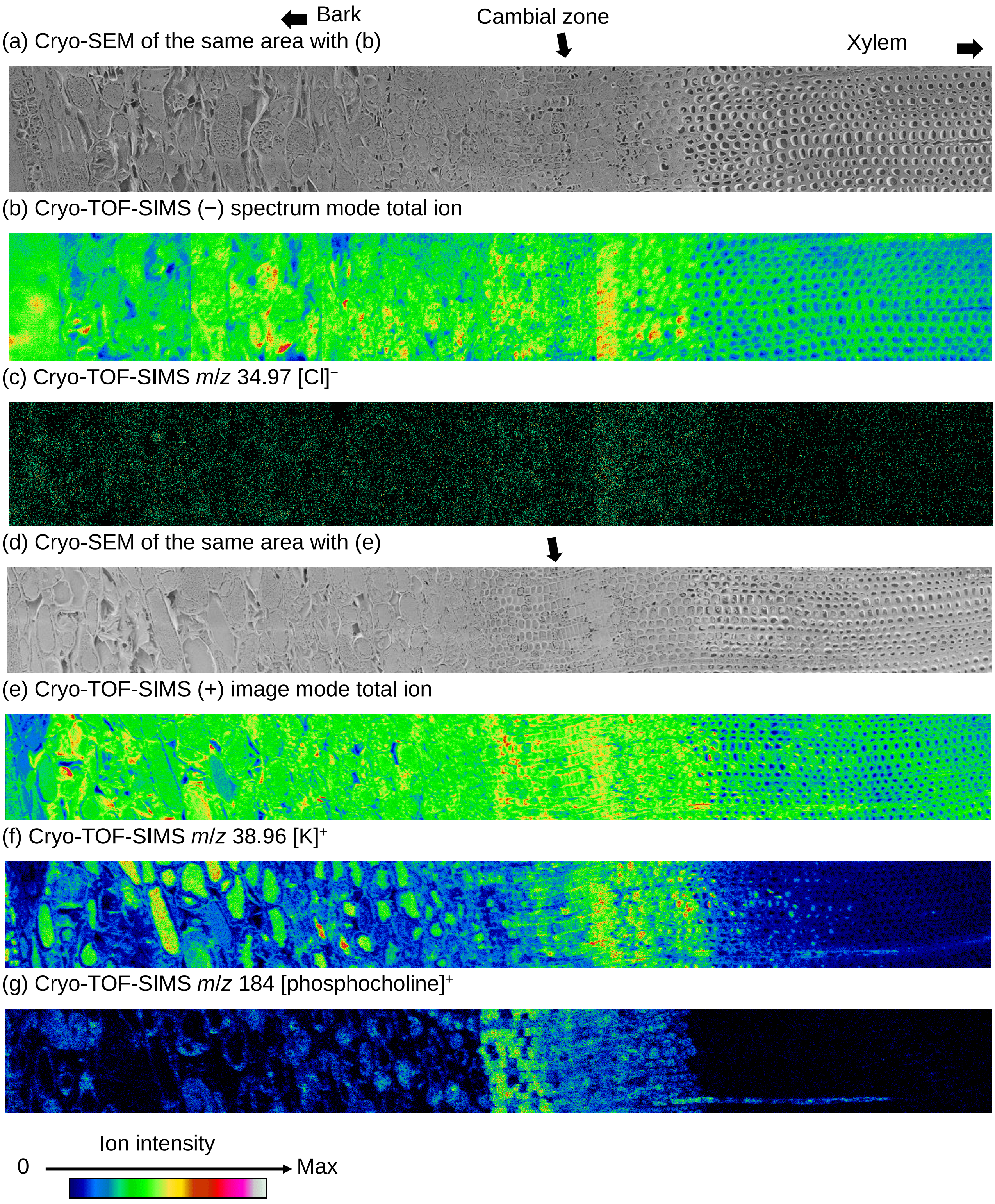

Fig. S7 Images of (a,d) cryo-SEM, (b,e) cryo-TOF-SIMS total ion, (c) Cl^-, (f) K^+, and (g) $[phosphocholine]^+$ of transverse surfaces of freeze-fixed *P. thunbergii* compression wood.

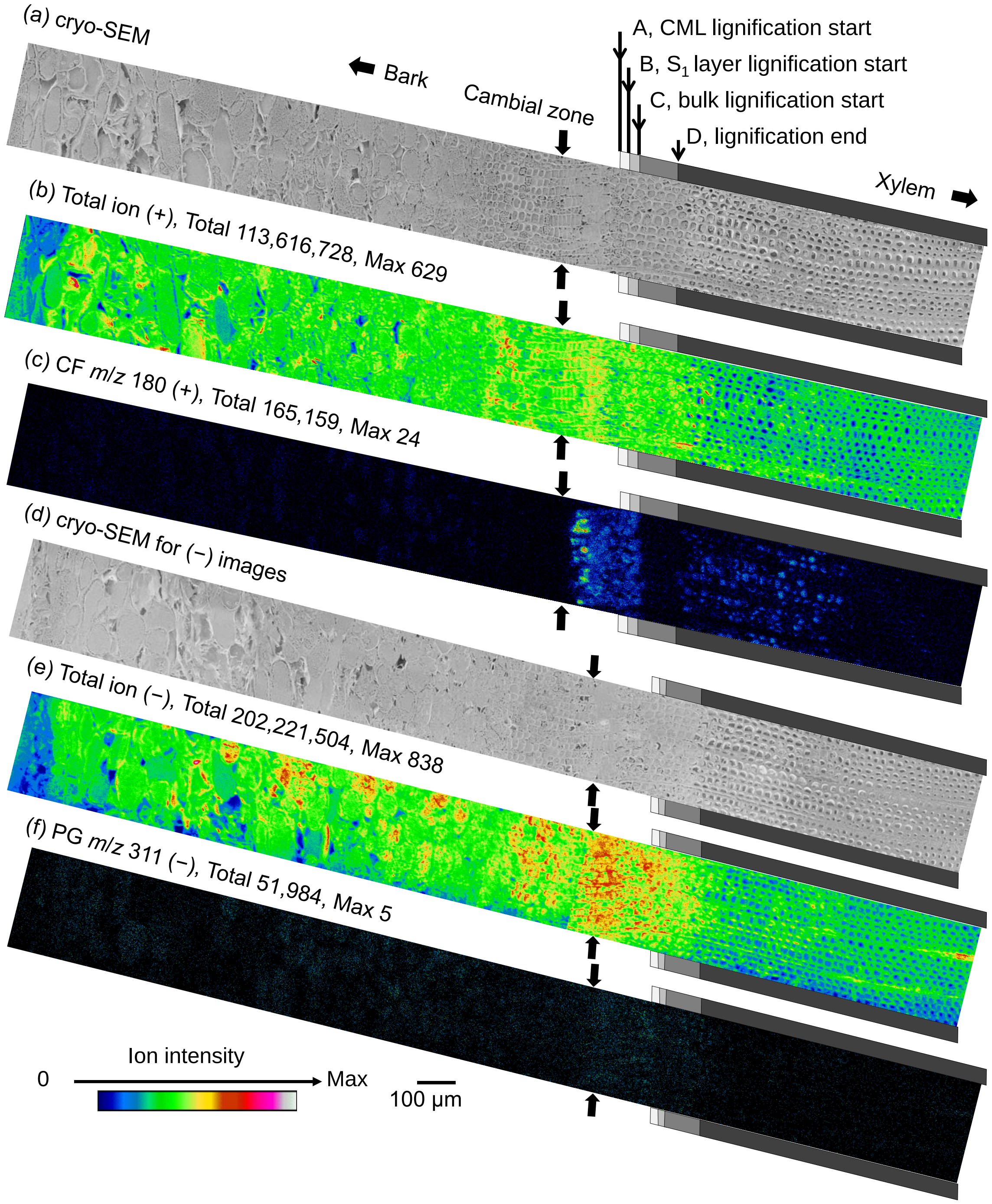

Fig. S8 Rotated cryo-TOF-SIMS/SEM images of the frozen-hydrated transverse surfaces of *P. thunbergii* compression wood: (a) cryo-SEM of the same region of (b), (b) total ion (+), (c) CF *m/z* 180 (+) ion, (d) cryo-SEM of the same region of (e), (e) total ion (−), and (f) PG *m/z* 311 (−) ion. Rotation angles were 12 ° for positive and 14 ° for negative images. The lignification stages determined by microscopic observations are suggested by the grey scaled tetragons. The scale bar is 100 µm. The cell wall lignification (lig.) stages of tracheids are suggested by A, B, C, and D by the same manner in Fig. 4.

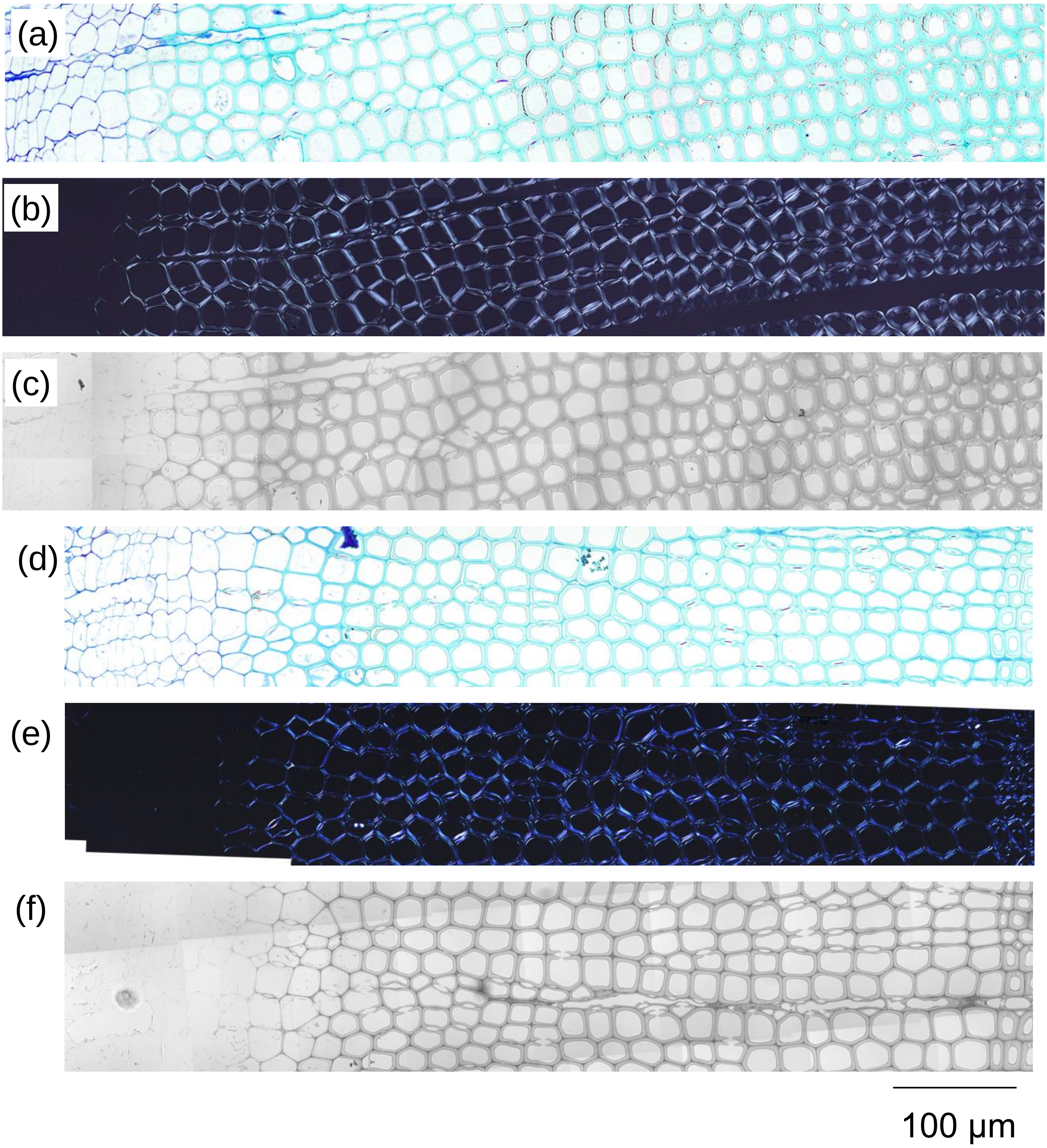

Fig. S9 A part of microscopic images for (a, b, c) compression and (d, e, f) opposite wood samples; by (a, d) saining and optical microscopy, (b, e) polarized optical microscopy, and (c, f) UV microscopy. By polarized optical microscopy, S3 layer absence can be checked as a result of the compression wood formation. By UV microscopy, S2L layer is visualized within S1 and S2 layers in compression wood.

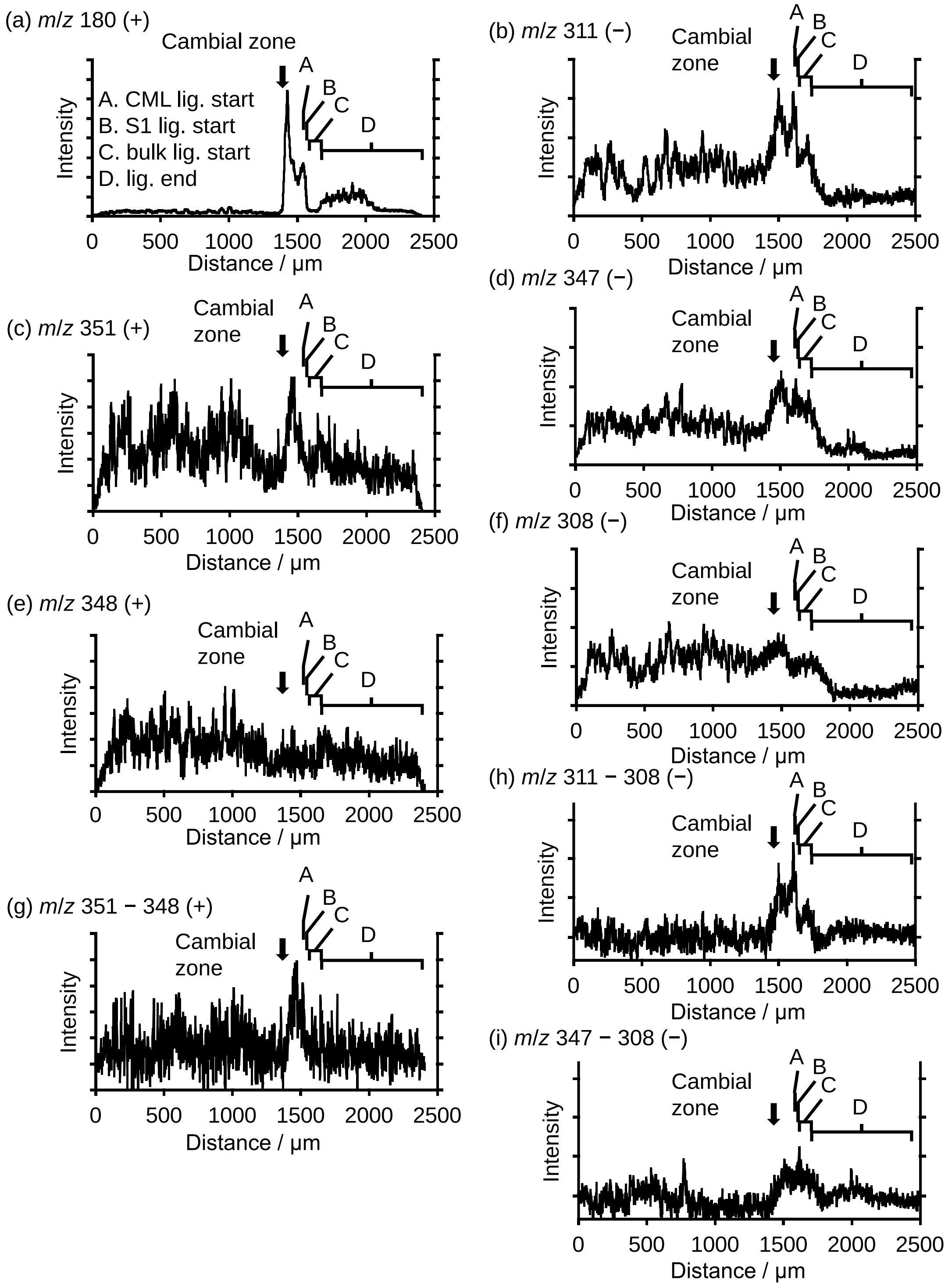

Fig. S10 Radial distribution of cryo-TOF-SIMS (a,c,e) positive and (b,d,f) negative ions. For positive ions, (a) *m*/*z* 180 ion, (c) *m*/*z* 351 ion, and (e) *m*/*z* 348 ion as a background candidate. (d) The differential ion count profile between *m*/*z* 351 and 348 ions. For negative ions, (b) *m*/*z* 311 ion, (d) *m*/*z* 347 ion, and (f) *m*/*z* 308 ion as a background. Differential ion count profiles for *m*/*z* 311 and 347 ions with *m*/*z* 308 ion are shown in (h) and (i), respectively. Line profiles were obtained after image rotation of 12 ° for positive and 14 ° for negative images, as shown in Fig. S8. The cell wall lignification (lig.) stages of tracheids are suggested by A, B, C, and D by the same manner in Fig. 4.

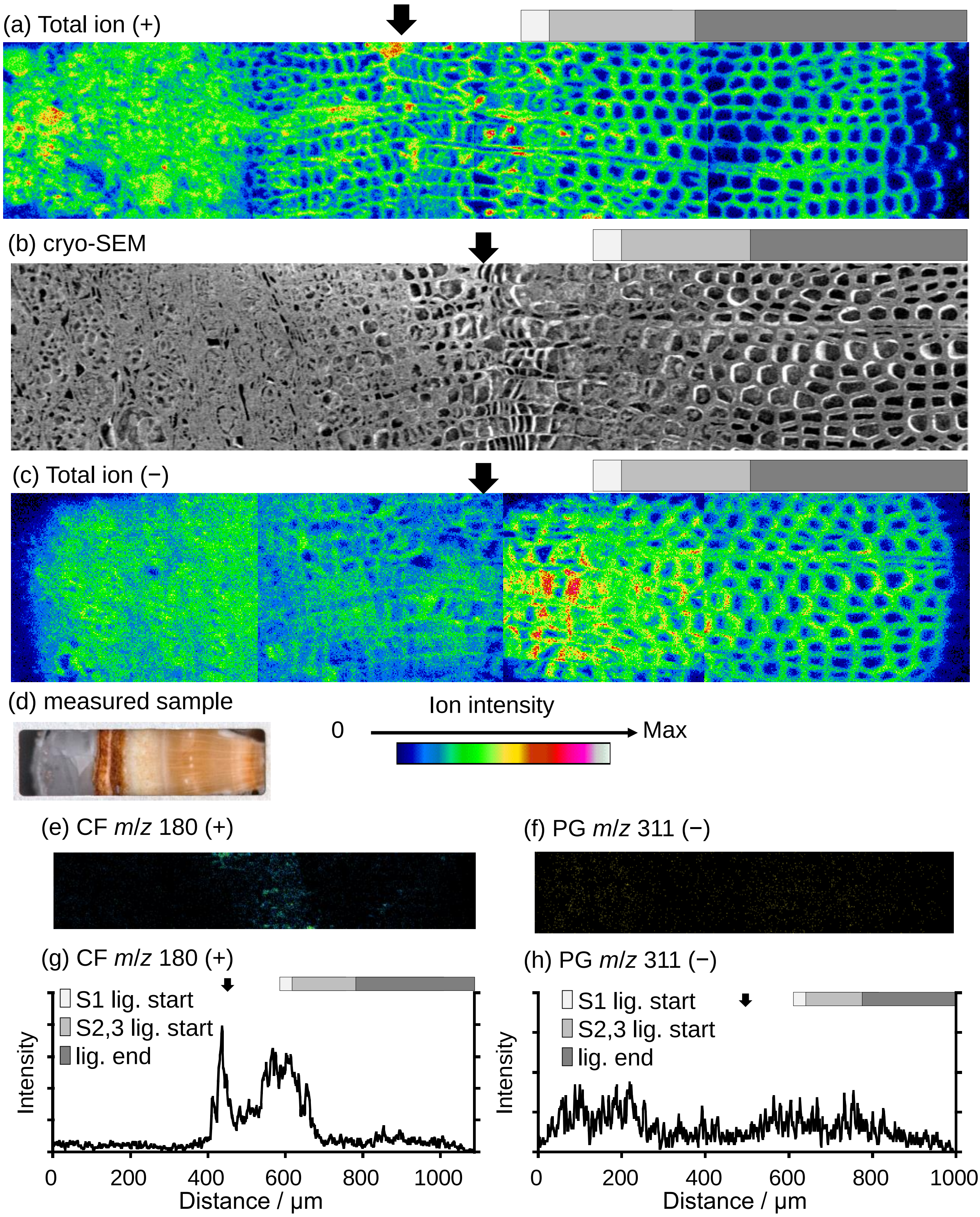

Fig. S11 Cryo-TOF-SIMS/SEM images obtained for the frozen-hydrated transverse surfaces of *P. thunbergii* opposite wood: total ion (+, a; −, c), (b) cryo- SEM of the same region of (c), (e) *m*/*z* 180 ion and (f) 311 ion, radial distribution of (g) *m*/*z* 180 ion count and (h) *m*/*z* 311 ion count. The lignification stages determined by microscopic observations are suggested by the grey scaled tetragons.

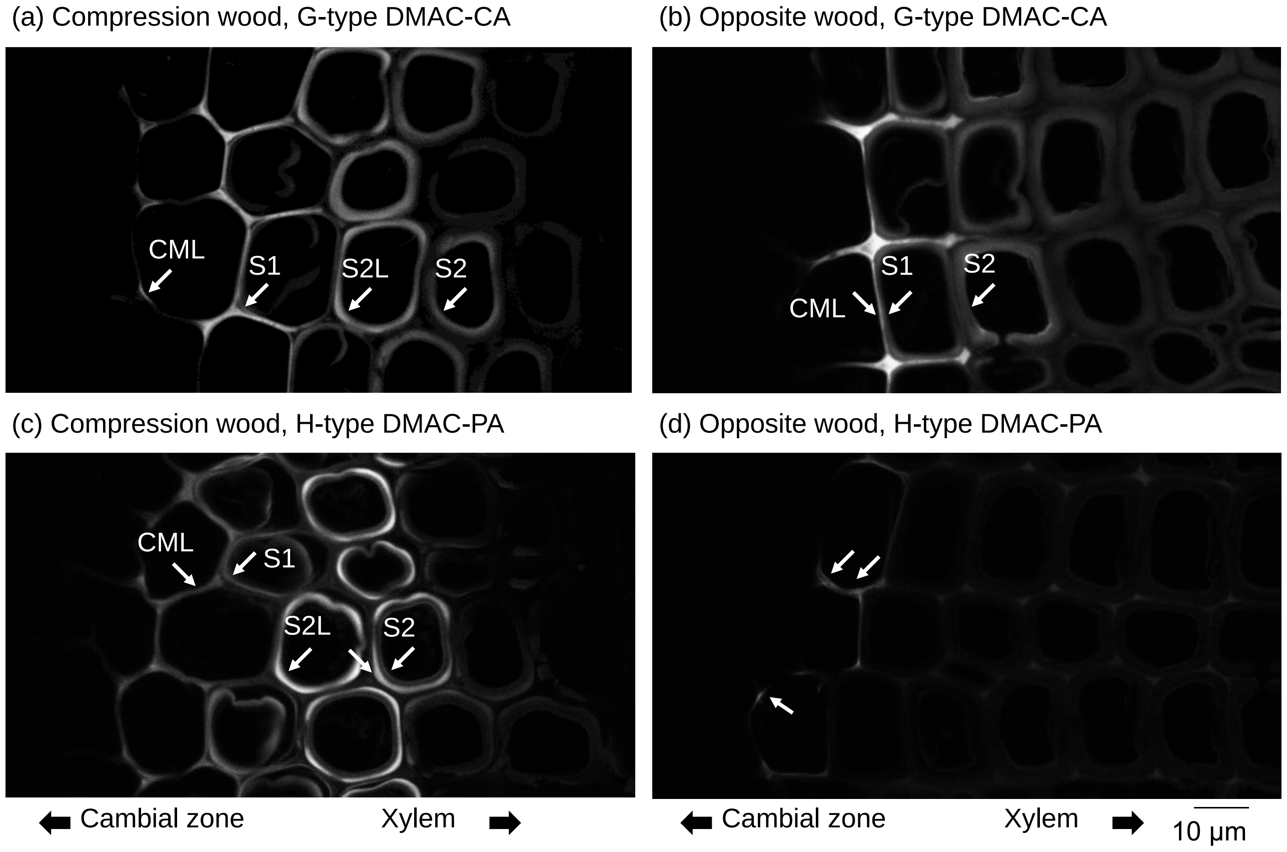

Fig. S12 Enlarged images of oxidative enzyme depending fluorescence-tagged ML incorporation in the differentiating xylem region of (a,c) compression and (b,d) opposite wood of *P. thunbergii*. The scale bar is 10 μm.

Histochemical staining method

Phloroglucinol-HCl (Kim et al., 2010, Hiraide et al. 2021) and 3,3′-diaminobenzidine (DAB) (Hiraide et al., 2016, 2021; Ranocha et al., 1999) stainings were performed as described previously.

References

Hiraide, H., Yoshida, M., Sato, S., and Yamamoto, H. (2016) In situ detection of laccase activity and immunolocalisation of a compression-wood-specific laccase (CoLac1) in differentiating xylem of Chamaecyparis obtusa. Functional Plant Biology 43:542–552.

Hiraide, H., Tobimatsu, Y., Yoshinaga, A., Lam, P.Y., Kobayashi, M., Matsushita, Y., Fukushima, K., and Takabe K. (2021) Localised laccase activity modulates distributions of lignin polymers in gymnosperm compression wood. New Phytologist doi: 10.1111/nph.17264.

Kim, J.S., Awano, T., Yoshinaga, A., and Takabe, K. (2010) Immunolocalization of β-1-4-galactan and its relationship with lignin distribution in developing compression wood of Cryptomeria japonica. Planta 232:109–119.

Ranocha, P., McDougall, G., Hawkins, S., Sterjiades, R., Borderies, G., Stewart, D., Cabanes-Macheteau, M., Boudet, A.M., and Goffner, D. (1999) Biochemical characterization, molecular cloning and expression of laccases - a divergent gene family - in poplar. European Journal of Biochemistry 259:485–495.
